# SICA-mediated cytoadhesion of *Plasmodium knowlesi*-infected red blood cells to human umbilical vein endothelial cells

**DOI:** 10.1038/s41598-022-19199-0

**Published:** 2022-09-02

**Authors:** Huai Chuang, Miako Sakaguchi, Amuza Byaruhanga Lucky, Junya Yamagishi, Yuko Katakai, Satoru Kawai, Osamu Kaneko

**Affiliations:** 1 Department of Protozoology, Institute of Tropical Medicine (NEKKEN), Nagasaki University, 1-12-4 Sakamoto, Nagasaki, 852-8523 Japan; 2 Leading Program, Graduate School of Biomedical Sciences, Nagasaki University, Nagasaki, Japan; 3 Central Laboratory, Institute of Tropical Medicine (NEKKEN), Nagasaki University, 1-12-4 Sakamoto, Nagasaki, 852-8523 Japan; 4 International Institute for Zoonosis Control, Hokkaido University, Sapporo, Japan; 5 The Corporation for Production and Research of Laboratory Primates, Tsukuba, Ibaraki Japan; 6 Department of Tropical Medicine and Parasitology, Dokkyo Medical University, Tochigi, Japan; 7 Present Address: Department of Internal Medicine, Morsani College of Medicine, University of South Florida, Tampa, FL USA

**Keywords:** Parasitology, Parasite biology

## Abstract

Zoonotic malaria due to *Plasmodium knowlesi* infection in Southeast Asia is sometimes life-threatening. Post-mortem examination of human *knowlesi* malaria cases showed sequestration of *P. knowlesi*-infected red blood cells (iRBCs) in blood vessels, which has been proposed to be linked to disease severity. This sequestration is likely mediated by the cytoadhesion of parasite-iRBCs to vascular endothelial cells; however, the responsible parasite ligands remain undetermined. This study selected *P. knowlesi* lines with increased iRBC cytoadhesion activity by repeated panning against human umbilical vein endothelial cells (HUVECs). Transcriptome analysis revealed that the transcript level of one gene, encoding a Schizont Infected Cell Agglutination (SICA) protein, herein termed SICA-HUVEC, was more than 100-fold increased after the panning. Transcripts of other *P. knowlesi* proteins were also significantly increased, such as PIR proteins exported to the iRBC cytosol, suggesting their potential role in increasing cytoadhesion activity. Transgenic *P. knowlesi* parasites expressing Myc-fused SICA-HUVEC increased cytoadhesion activity following infection of monkey as well as human RBCs, confirming that SICA-HUVEC conveys activity to bind to HUVECs.

## Introduction

A global public health strategy was implemented to end malaria epidemics by 2030; however, malaria still resulted in 241 million estimated cases and 627,000 deaths in the world in 2020^1^. In addition to four *Plasmodium* species transmitted among humans, a zoonotic infection of the simian malaria parasite *Plasmodium knowlesi* was recognized in 2004 following an epidemic in a human community in West Malaysia^2^. Since then, human infections with *P. knowlesi* have been reported from other Southeast Asian countries such as Indonesia, Myanmar, Laos, Singapore, Thailand, Philippines, Cambodia, and Vietnam^3^. *P. knowlesi* is transmitted to humans by mosquitoes previously fed on infected monkeys, whereas human-mosquito-human transmission has not been definitively identified. Increased deforestation and anthropogenic land use have been suggested to lead to close contact among mosquito vectors, primary host monkeys, and potential host humans^4^. Thus *P. knowlesi* is now regarded by the WHO as one of the causative agents of human malaria^1^.

Compared to the other human malaria parasites that require at least 2 days to complete one cycle of intraerythrocytic replication, *P. knowlesi* progresses through the asexual blood cycle in 24 h. Thus the parasite can relatively rapidly reach high bloodstream parasitemias, which are thought to be related to disease severity^5^. Deaths due to *knowlesi* malaria are increasingly seen, especially in Sabah state, Malaysian Borneo^6^. In *Plasmodium falciparum*, asexual parasites sequestered from the peripheral blood can provoke severe pathologies, such as cerebral malaria neurological symptoms, characterized by coma and oftentimes death^7^. *P. falciparum* sequestration is mediated by a parasite ligand, called *Plasmodium falciparum* erythrocyte membrane protein 1 (PfEMP1), which is displayed on the surface of infected red blood cells (iRBCs) and confers adhesion to vascular endothelial cells. Sequestration prevents the clearance of iRBCs by the spleen and serves as a major virulence factor^8^. Sequestration of *P. knowlesi*-iRBCs in blood vessels was observed in a post-mortem examination of humans and monkeys^9,10^. Abdominal pain was found to be a risk factor for severe *knowlesi* malaria^11^, for which gut ischemia due to accumulation of iRBCs in the blood vessels was suggested as a cause^6^. Schizont Infected Cell Agglutination (SICA) proteins have been proposed to have a role in the cytoadhesion of *P. knowlesi*-iRBCs^12^; however, this interaction is not well characterized. SICA protein is a type I transmembrane protein encoded by a *SICAvar* multigene family with at least 136 members^13^. This protein has multiple cysteine-rich domains (CRDs) in the extracellular region, and an intracellular region which shares homology with *Plasmodium* molecules expressed on the RBC membrane, such as *P. falciparum* PfEMP1 and SURFIN family members^12,14^. In addition to SICA protein, *P. knowlesi* expresses KIR proteins, encoded by the *pir* gene family which is widely represented in the rodent and primate malaria parasites, and for which binding activity of the *P. vivax* homolog VIR was reported^15^.

To gain insights into the mechanism of sequestration in *P. knowlesi*-iRBCs in humans, we aimed to identify molecules responsible for the cytoadhesion of *P. knowlesi*-iRBCs to human endothelial cells. We repeatedly panned *P. knowlesi*-iRBCs against human venous endothelial cells to enrich cytoadhesive iRBCs. RNA-seq analysis of parasites with cytoadherence activity identified one *SICA* open reading frame (ORF) as a strong candidate, and following a transfection experiment confirmed that the identified SICA was capable of increasing the cytoadhesive activity of transfected parasites.

## Results

### Cytoadhesion of *P. knowlesi*-iRBCs against human umbilical vein endothelial cells (HUVECs) is unstable

**Figure 1:**
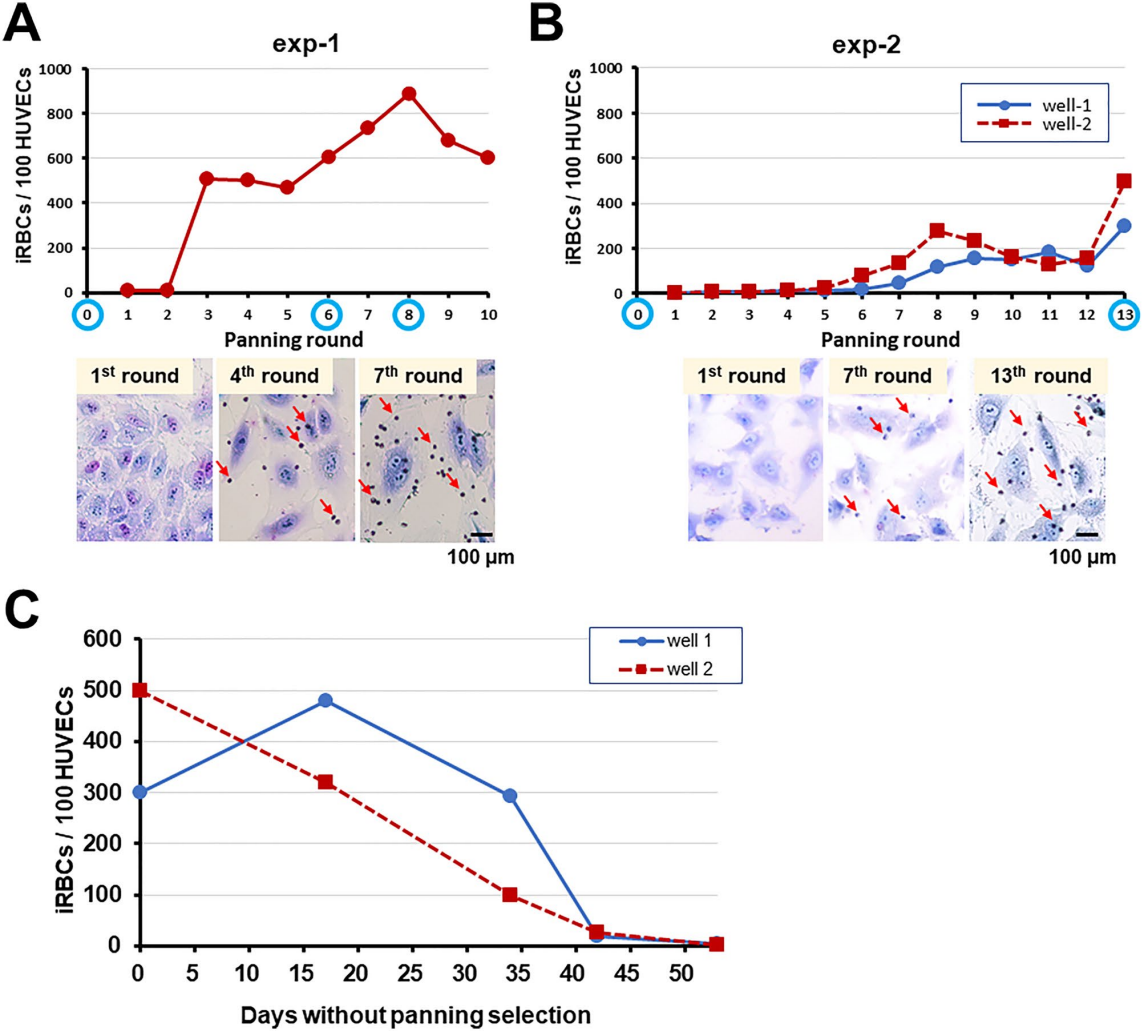
Panning selection of *P. knowlesi*-iRBCs against HUVECs. (**A**) First panning selection experiment (exp-1). RNA samples were obtained before and after the 6th and 8th pans (blue circle on the x-axis). Images after the 1st, 4th, and 7th rounds are shown below. (**B**) Second panning selection experiment (exp-2). Two wells (well-1 and well-2) were simultaneously panned 13 times and RNA samples are collected before and after the 13th pan (blue circle on the x-axis). Images after the 1st, 7th, and 13th rounds are shown below. (**C**) Binding activity of iRBCs in 2 wells at 17, 34, 42, and 53 days after the 13th pan of exp-2 without panning selection pressure.

To evaluate if *P. knowlesi*-iRBCs could bind to human endothelial cells, we repeatedly panned monkey RBCs infected with a wild-type *P. knowlesi* on HUVECs. The first selection experiment (exp-1) was initiated with two wells, but one well was contaminated; thus, only one sample before the panning selection (“pre-1” sample) was used to compare with samples after the 6th and 8th pans. Bound iRBCs were not detected at the beginning, but we observed iRBCs bound on the HUVECs from the 3rd pan onward, and with each panning the number of bound RBCs gradually increased (Fig. 1A). RNA samples were obtained before (“pre-1”) and after the 6th and 8th pans from one well (“6th pan” and “8th pan”, respectively). When the culture was maintained without panning selection for 88 days after the 10th pan, the binding activity became undetectable. The increased binding activity was repeatedly observed when the culture was panned, which was reduced if the panning selection was not performed (Fig. S9A). For the second selection experiment (exp-2), 2 wells were simultaneously panned against HUVECs, and bound iRBCs were seen after the 5th pan and the number of bound RBCs increased with each panning (Fig. 1B). RNA samples were prepared before (“pre-3”) and after the 13th pan (“well-1” and “well-2”). After the 13th pan, we further maintained these 2 parasite lineages without panning selection and examined their cytoadhesion activity on days 17, 34, 42, and 53 after the 13th pan. We found that the cytoadhesion activity of the two lineages gradually decreased and the level became similar to the level before panning selection on day 53 after the 13th pan was performed (Fig. 1C). These results suggest that the cytoadhesion property of *P. knowlesi*-iRBCs was unstable.

### Transcripts of two *SICAvar* fragments significantly and reproducibly increased after panning selections

**Figure 2:**
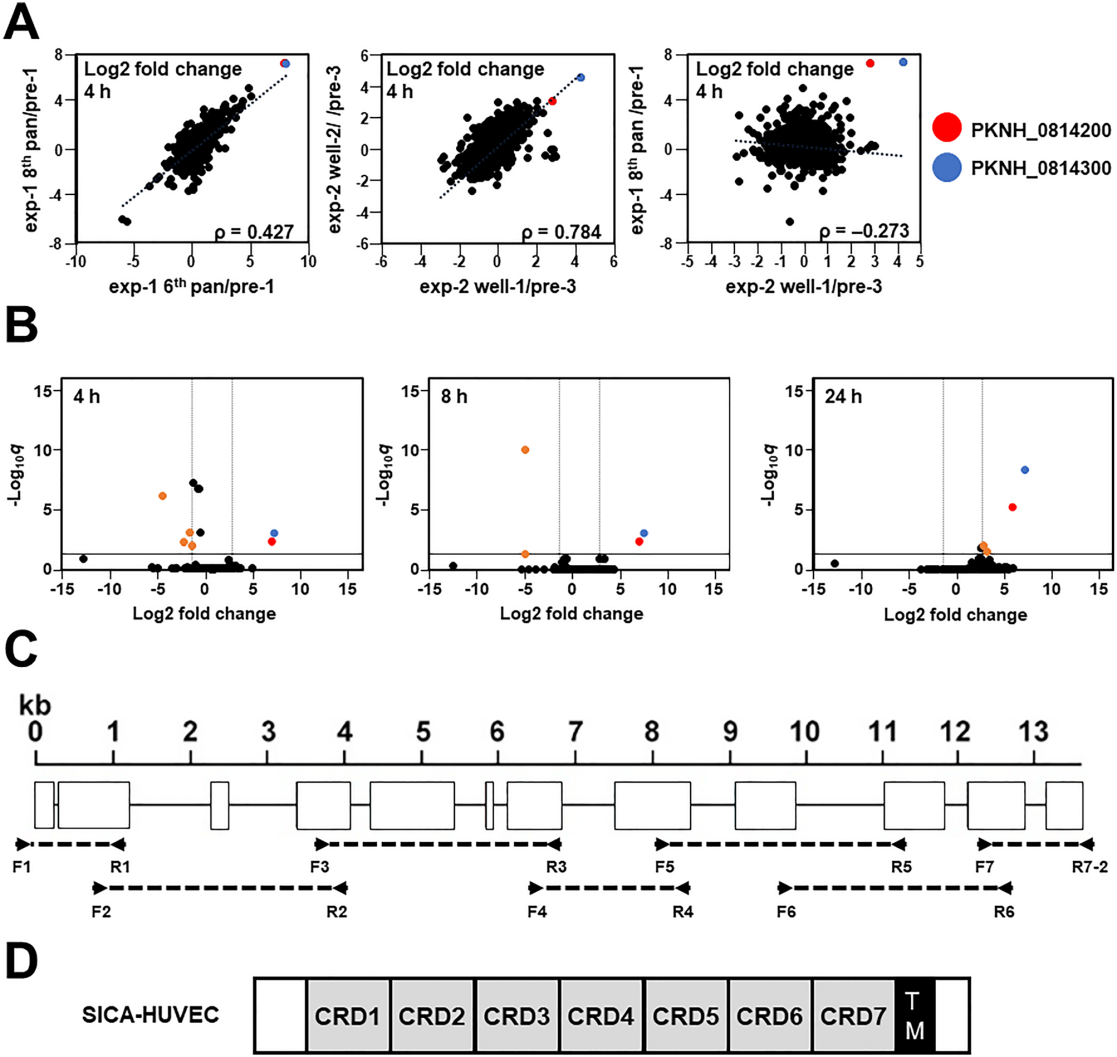
RNA-seq data analysis of cytoadherent and wild-type *P. knowlesi*. (**A**) Correlation of the fold change of the transcript levels between samples at 4 h after RBC invasion. The coordinate axes show log2 fold changes of transcript amounts by panning selection of the 6th and 8th pans of exp-1 (left), wells 1 and 2 samples of exp-2 13th pan (middle), and samples after the 8th pan of exp-1 and well-1 of exp-2 13th pan (right). Spearman's rank correlation coefficients (ρ) are shown. (**B**) Volcano plots showing log2 fold changes of transcript amounts by panning selection plotted against the − log_10_ of *q* value obtained by the comparison between non-binding control samples (pre-1, -2, and -3) and binding parasites samples (8th pan of exp-1, wells 1 and 2 of exp-2 13th pan) at three different time points. The dashed and solid lines indicate absolute log2 fold change = 2 and *q* = 0.05, respectively. Genes significantly differentially expressed (*q* < 0.05) and at least fourfold changed are shown in orange dots. Genes with non-significant differences or less than fourfold changed are shown in black dots. Gene fragments of PKNH_0814200 and PKNH_0814300 are highlighted in red and blue, respectively. (**C**) A schematic depiction of the *sica-huvec* exon–intron structure, which contains 12 exons. Locations are indicated for the primers used to amplify the fragments to determine the cDNA sequence. (**D**) Predicted domains of SICA-HUVEC. CRD, cysteine-rich domain; TM, transmembrane domain. The size is not to scale.

RNA samples were collected at 4 h (ring form early trophozoite), 8 h (late trophozoite), and 24 h (schizont) after RBC invasion and were subjected to RNA-seq analysis. The transcript expression fold change was obtained by dividing the values after panning by the values before panning. Firstly, we assessed the correlation among samples. The scatter plot of the log2 fold change between the 6th and 8th pans of exp-1 against pre-1 revealed that these two samples were positively correlated with a moderate ρ value (0.427) (Fig. 2A, left). A positive correlation was also observed between wells 1 and 2 of the exp-2 13th pan against pre-3 (ρ = 0.784, Fig. 2A, middle). However, there was no positive correlation between exp-1 and exp-2 (Fig. 2A, right for exp-1 8th pan against pre-1 and exp-2 13th pan well-1 against pre-3; Figs. S1 and S2 for other combinations), suggesting that if the culture was handled simultaneously, the transcription pattern was similarly changed, but if the culture process was independent, the change of the transcription pattern differed. This observed difference between exp-1 and exp-2 is not due to the inclusion of the ORFs with low coverage, because the analysis excluding ORFs for which the average FPKM of all data was less than 10 also showed the same tendency (Figs. S3 and S4). The independent panning selections both identified two ORFs, PKNH_0814200 (type II SICA) and PKNH_0814300 (type I SICA), that increased their transcript expression (Fig. 2A, red and blue dots, respectively).

Data from the 8th pan of exp-1 and the 2 wells (well-1 and well-2) of exp-2 13th pan were combined, and the log2 fold change of each ORF and the significance (− log_10_ *q*) were visualized by volcano plots (Fig. 2B). The data of the exp-1 6th pan was excluded because the samples after the 6th and 8th pans were collected from the same culture lineage. Volcano plots indicated that PKNH_0814200 and PKNH_0814300 described above were the most differentially and highly expressed ORFs at all 3-time points. Tables 1 and 2 summarize ORFs for which the average FPKM of all data was at least 10, the mRNA expression was at least twofold increased (Table 1) or decreased (Table 2) after the panning by the analysis of the combined data, *q* value < 0.05, and consistently increased or decreased in all 4 test samples (exp-1 6th and 8th pans and exp-2 13th pan wells 1 and 2). In addition to PKNH_0814200 and PKNH_0814300, the transcripts of 2 PIR proteins (PKNH_0312000 and PKNH_0807800), which are predicted to be exported to the RBC cytosol, were increased consistently throughout the samples at the schizont stage (24 h). Consistently decreased ORFs included 3 SICA-related proteins (PKNH_1144600, PKNH_0727400, and PKNH_1144500) and proteins exported to the RBC cytosol (PKNH_1401700 and PKNH_1357100). When a less stringent criteria was used with a *P* value less than 0.05, 16 differentially expressed ORFs with absolute log2 fold change > 2 and *P* value < 0.05 at 4 h, 8 ORFs at 8 h, and 50 ORFs at 24 h were identified which included more SICA members and PIR proteins (Tables S2 and S3, Figs. S5 and S6).

**Table 1:** Identified *P. knowlesi* ORFs whose transcript expression was significantly increased.

ID	Annotation	Log2 fold change after panning				*P* value	*q* value
		exp-1		exp-2			
		6th pan	8th pan	well-1	well-2		
**4 h (ring form early trophozoite)**							
PKNH_0814300	SICA, type I (fragment)	8.05	7.27	4.29	4.60	1.34 × 10^−6^	8.93 × 10^−4^
PKNH_0814200	SICA, type II (fragment)	7.87	7.19	2.84	3.09	6.88 × 10^−6^	4.01 × 10^−3^
**8 h (late trophozoite)**							
PKNH_0814300	SICA, type I (fragment)	8.81	8.19	4.03	4.02	3.20 × 10^−7^	8.26 × 10^−4^
PKNH_0814200	SICA, type II (fragment)	8.03	7.51	3.13	3.27	2.63 × 10^−6^	4.53 × 10^−3^
**24 h (schizont)**							
PKNH_0814300	SICA, type I (fragment)	8.05	7.74	3.91	3.70	8.61 × 10^−13^	4.59 × 10^−9^
PKNH_0814200	SICA, type II (fragment)	8.09	7.53	2.76	2.22	2.13 × 10^−9^	5.67 × 10^−6^
PKNH_0312000	PIR protein	0.84	1.53	2.18	1.83	5.21 × 10^−6^	9.25 × 10^−3^
PKNH_0807800	PIR protein	2.33	2.52	1.70	1.58	3.32 × 10^−5^	0.0354

ORFs satisfying the following criteria are shown: (1) whose average FPKM of all data was at least 10, (2) average fold change values were at least 4, and (3) *q* value is less than 0.05. *P* and *q* values were obtained excluding the exp-1 6th pan data.

**Table 2:** Identified *P. knowlesi* ORFs whose transcript expression was significantly decreased.

ID	Annotation	Log2 fold change after panning				*P* value	*q* value
		exp-1		exp-2			
		6th pan	8th pan	well-1	well-2		
**4 h (ring form early trophozoite)**							
PKNH_1144600	SICA, type II	− 5.54	− 6.19	− 0.59	− 0.49	5.47 × 10^−10^	6.38 × 10^−7^
PKNH_0727400	SICA, type I	− 1.31	− 1.87	− 0.94	− 0.95	1.26 × 10^−6^	8.93 × 10^−4^
PKNH_1401700	*Plasmodium* exported protein	− 3.60	− 3.16	− 0.40	− 0.25	1.01 × 10^−5^	5.24 × 10^−3^
PKNH_1357100	*Plasmodium* exported protein	− 3.04	− 2.56	− 0.22	− 0.12	2.11 × 10^−5^	9.84 × 10^−3^
**8 h (late trophozoite)**							
PKNH_1144600	SICA, type II	− 5.49	− 6.47	− 1.62	− 1.07	1.82 × 10^−14^	9.40 × 10^−11^
PKNH_1144500	SICA fragment	− 5.41	− 5.10	− 1.24	− 1.45	3.83 × 10^−5^	0.0494
**24 h (schizont)**							
None satisfied							

ORFs satisfying the following criteria are shown: (1) whose average FPKM of all data was at least 10, (2) average fold change values were less than or equal to 4, and (3) *q* value is less than 0.05. *P* and *q* values were obtained excluding the exp-1 6th pan data. PKNH_1144500 was annotated as “Protein conserved in *P. knowlesi*” in PlasmoDB, but identified as a SICA fragment by BLASTP analysis.

We then evaluated whether ORFs whose transcript levels increased with the increase in cytoadhesion activity would decrease with the loss of adhesion activity. To this end, we conducted RNA-seq analysis against *P. knowlesi* samples (both well-1 and well-2) at 53 days after the 13th pan in experiment 2 described above. Samples from both wells showed that FPKM values of PKNH_0814200 and PKNH_0814300 decreased at all three time points (Table S4). In addition, we found increased FPKM values of PKNH_0312000 and PKNH_0807800 (both are PIR proteins) with increasing cytoadhesion activity decreased with loss of the adhesion activity; whereas decreased FPKM values of PKNH_1144500 and PKNH_1144600 (both are SICA proteins) with increasing cytoadhesion activity increased with loss of the adhesion activity.

### Two *SICAvar* fragments annotated in PlasmoDB belong to one ORF frame

We focused on ORFs PKNH_0814200 and PKNH_0814300 because they were changed most highly and significantly compared to the other ORFs. Examination of the genome sequence information of *P. knowlesi* H strain in the PlasmoDB database revealed that PKNH_0814200 and PKNH_0814300 are adjacent within the genome, but sequence between the two ORFs is lacking. Because PKNH_0814300 encodes the N-terminal side and PKNH_0814200 encodes the C-terminal side of a predicted SICA protein, we evaluated the genome information for another line of *P. knowlesi* (Malayan strain PK1 A) in PlasmoDB and found that the two H strain ORFs are annotated as a single ORF (PKNOH_S100042200). To this end, we determined the cDNA sequence in the H strain and found that PKNH_0814200 and PKNH_0814300 indeed represent one ORF consisting of 12 exons (Fig. 2C). We named this gene *sica-huvec* and the encoded protein SICA-HUVEC. Experimentally obtained cDNA sequence was deposited into the DDBJ database (LC663824). SICA-HUVEC is a type I transmembrane protein composed of one conserved head domain and seven SICA cysteine-rich domains in its extracellular region, followed by one transmembrane domain and a cytoplasmic region (Fig. 2D).

### Establishment of a transgenic *P. knowlesi* parasite line expressing SICA-HUVEC-Myc

**Figure 3:**
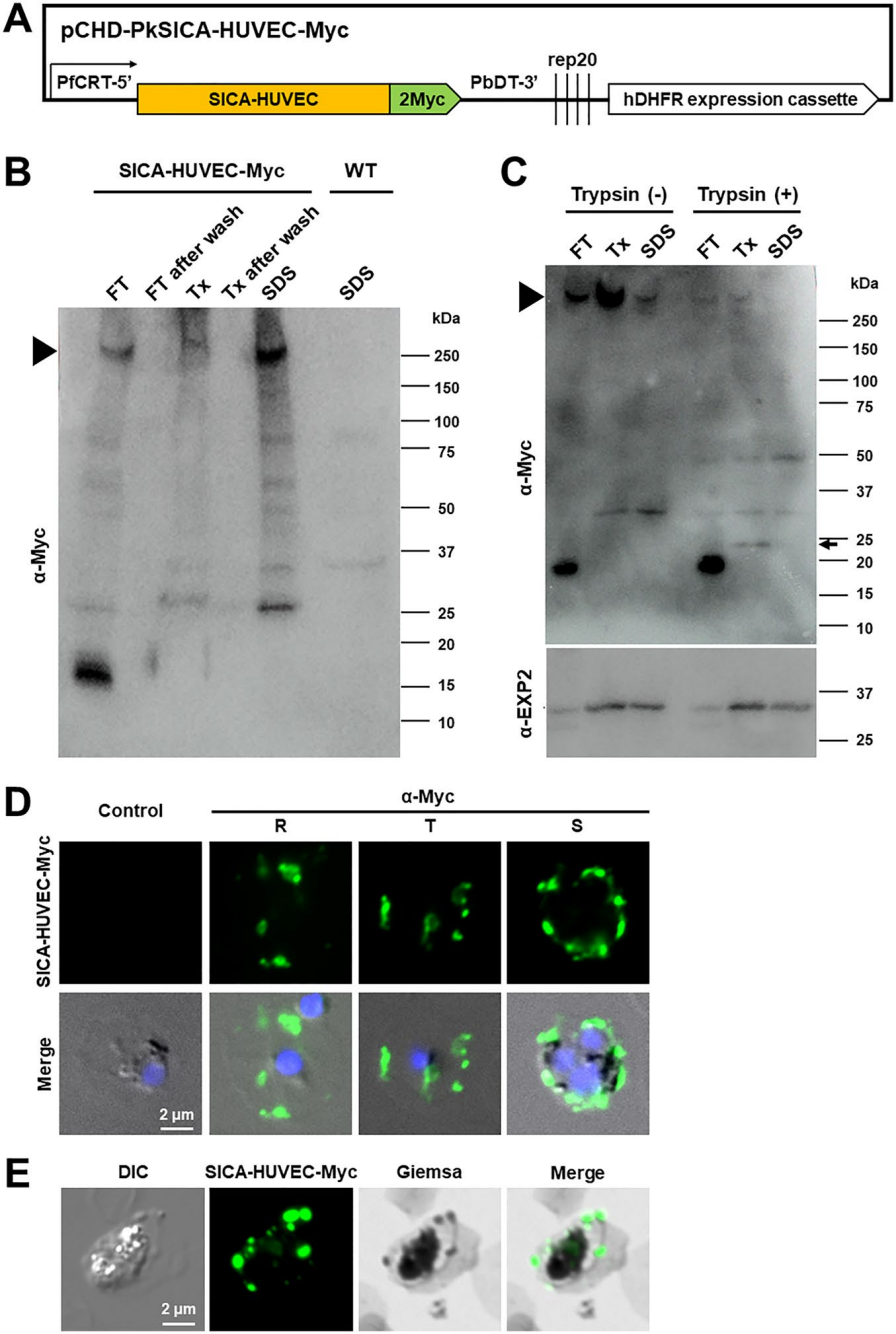
Generation of a transgenic *P. knowlesi* line expressing SICA-HUVEC-Myc using monkey RBCs. (**A**) A schematic image of the SICA-HUVEC-Myc expression construct. Full-length of SICA-HUVEC was fused with Myc epitopes (Myc) at its C terminus and the *P. falciparum* CRT promoter region (PfCRT 5′) was used as a promoter. A human DHFR expression cassette was used for the drug selection. (**B**) Western blotting of the wild-type parental *P. knowlesi* line (WT) and the transgenic line expressing SICA-HUVEC-Myc in monkey RBCs. Proteins were sequentially extracted by freeze-thawing (FT), with 1% Triton X-100 (Tx), and then with 2% SDS for the transfectants; whereas they were extracted with 2% SDS only for the wild type parasites. Bands detected with anti-Myc antibody (α-Myc) around the expected size for the SICA-HUVEC-Myc are indicated with an arrowhead. (**C**) Trypsin treatment of the transgenic line expressing SICA-HUVEC-Myc. Trypsin-treated or untreated samples were sequentially extracted as described above and subjected to Western blot with α-Myc or a loading control anti-EXP2 antibody (α-EXP2; The original blot is presented in Fig. S10). To make the 24-kDa band (arrow) easier to see, the intensity of the upper image is increased. (**D**) IFA images of *P. knowlesi* expressing SICA-HUVEC-Myc in monkey RBCs stained with anti-Myc antibody (α-Myc, green). Signals detected with α-Myc were merged with DAPI nucleus signals (blue) and differential interference contrast (DIC) images (Merge). The bottom panels are images from a negative control reacted with normal mouse IgG. (**E**) Images of *P. knowlesi* expressing SICA-HUVEC-Myc in monkey RBCs stained with Giemsa's solution following IFA with α-Myc antibody. Signals detected with α-Myc were merged with Giemsa-stained images (Merge).

To validate if SICA-HUVEC was responsible for the cytoadhesion to HUVECs, a transgenic *P. knowlesi* line was established with an episomal plasmid expressing the full-length SICA-HUVEC fused with 2 Myc epitopes at its C-terminus (SICA-HUVEC-Myc) (Fig. 3A). To assess the expression of SICA-HUVEC-Myc, proteins were sequentially extracted from transfectant-infected monkey RBCs by repeated freeze–thaw (Fz); with a non-ionic detergent, Triton X-100 (Tx); and then an ionic detergent, SDS. Western immunoblotting with anti-Myc antibody revealed a major band of approximately 250 kDa in all Fz, Tx, and SDS fractions of the transfectant expressing SICA-HUVEC-Myc; which was similar to the calculated molecule weight of 230 kDa based on the amino acid sequence (Fig. 3B). SICA-HUVEC-Myc was not fully extracted with Tx, suggesting an association with detergent-resistant membrane; which is similar to the extraction pattern of *P. falciparum* PfEMP1. No band was detected from the SDS extract of the wild-type *P. knowlesi*, indicating that the bands observed for the transfectant were from exogenously expressed SICA-HUVEC-Myc protein.

When iRBCs were treated with trypsin, the intensity of the ~ 250-kDa band was significantly reduced and bands at approximately 50 kDa and 24 kDa were detected in the trypsin-treated sample, but not in the untreated sample (Fig. 3C). The size of the 24-kDa band was consistent with the expected size of the cleaved product after K_1876_ (25 kDa). The intensity of the control EXP2 protein was similar between trypsin-treated and untreated samples. These results suggest that SICA-HUVEC-Myc is exposed on the surface of iRBCs.

IFA with anti-Myc antibody yielded a dotted pattern within the iRBC cytosol for all parasite stages examined (Fig. 3D), consistent with the export of SICA-HUVEC-Myc and localization at Sinton Mulligan's clefts—membranous structures in the *P. knowlesi*-iRBC cytosol where SBP1 and MAHRP2 are localized^16,17^. Giemsa-staining after IFA confirmed SICA-HUVEC-Myc co-localized with Sinton Mulligan's stipplings (Fig. 3E). Although signals were not clearly seen on the iRBC membrane, the IFA results suggested trafficking of *Plasmodium* molecules destined for exposure on the iRBC membrane.

### Transgenic parasites expressing SICA-HUVEC-Myc in monkey, as well as human RBCs, showed higher cytoadhesion activity to HUVECs

**Figure 4:**
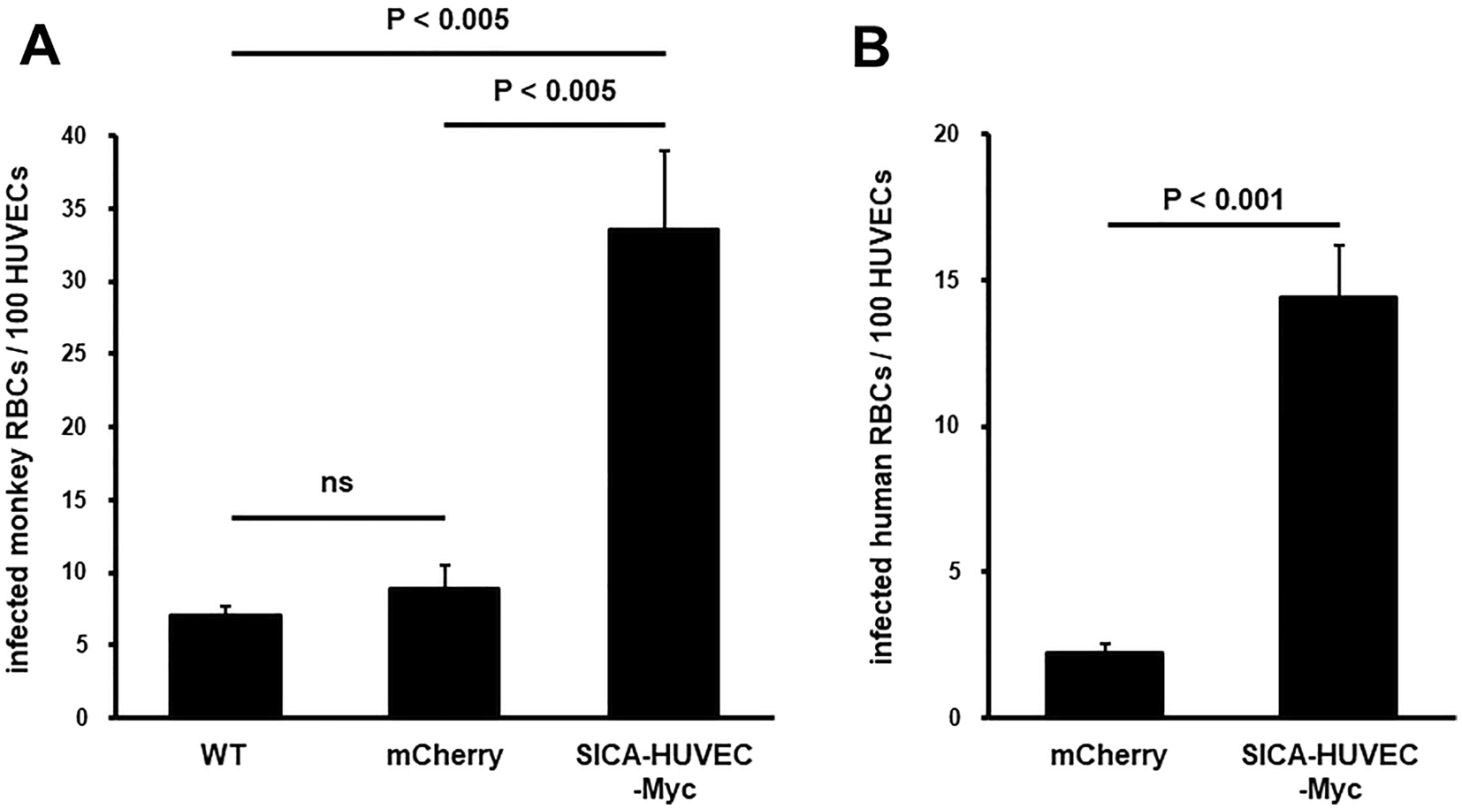
Cytoadhesion activity to HUVECs of monkey and human RBCs infected with transgenic parasites expressing SICA-HUVEC-Myc. (**A**) The number of infected monkey RBCs bound to HUVECs was compared among the parental wild-type parasite (WT), the transfectant expressing mCherry (mCherry), and the transfectant expressing SICA-HUVEC-Myc. (**B**) The number of infected human RBCs bound to HUVECs was compared between the transfectant expressing mCherry (mCherry) and the transfectant expressing SICA-HUVEC-Myc. Statistical differences were examined by Tukey's multiple comparison test after one-way ANOVA (**A**) or two-tailed Student's *t*-test (**B**). ns (not significant) indicates *P* > 0.05.

The HUVEC cytoadhesion activity was evaluated for RBCs infected with the obtained transfectant parasites. In addition to the wild-type parasite, a transfectant control was generated expressing mCherry in place of SICA-HUVEC-Myc (Fig. 4 and Fig. S7). Independent experiments using monkey RBCs consistently showed significantly higher binding of the SICA-HUVEC-Myc expressing transgenic parasite line than the two controls. The binding activity between the wild-type and mCherry-expressing transgenic parasites was not significantly different, excluding the possibility that transfection procedures affected the result (Fig. 4A,B). Together, these results indicated that *P. knowlesi* gained binding activity to HUVECs following the expression of SICA-HUVEC-Myc. We then evaluated if the cytoadhesion activity can be seen when human RBCs (hRBCs) were infected with the transfectant parasite line. Although long-term cultivation with hRBCs is not possible, the parental *P. knowlesi* H-DMU line can invade into and multiply within hRBCs for two cycles. We took advantage of this and let the purified schizonts of two transfectant lines invade into hRBCs, then the cytoadhesion activity of infected hRBCs was examined. We confirmed that approximately 95% of the prepared parasite-infected RBCs were hRBCs by differentiating hRBCs from monkey RBCs using an anti-human glycophorin A antibody (Fig. S8). Independent binding experiments using hRBCs consistently showed that the number of bound hRBCs infected with SICA-HUVEC-Myc-expressing parasites was significantly higher than that of hRBCs infected with mCherry-expressing parasites (Fig. 4B).

## Discussion

In this study, to the best of our knowledge we show for the first time that *P. knowlesi* parasites are able to change the cytoadhesion activity of their infected RBCs. We identified a SICA protein, termed SICA-HUVEC, which can mediate the cytoadhesion of HUVECs by infected monkey as well as human RBCs. Correspondingly, when iRBCs lost their cytoadhesion activity, the transcript level of SICA-HUVEC also decreased. In addition to SICA-HUVEC, RNA-seq analysis showed that transcripts for two PIR proteins were significantly increased in the cytoadherent parasites, suggesting that these proteins might be additively or synergistically involved in the cytoadhesion.

RBCs infected with transgenic *P. knowlesi* expressing Myc-tagged SICA-HUVEC did not bind as high as the naturally selected HUVEC-binding lines. qRT-PCR revealed that a similar amount of the transcripts for the putative ligand were detected from the transgenic line compared to the naturally selected parasites (Fig. S9B,C); indicating that the transcript levels were not the determinant of the difference in the cytoadhesion activity. Two possibilities can be considered; firstly, the introduced Myc-tagged SICA-HUVEC may not be exposed on the surface of iRBCs as efficiently as the natural SICA-HUVEC, perhaps due to the addition of the Myc-epitope to its C-terminus. The amino acid linker sequence between SICA-HUVEC and the Myc-epitopes is GTELLDGP. Glycine and proline are helix breaker residues that enable movement of the following domain freely from potential physical restriction by the upstream domain structure. We have used the same linker sequence to fuse Myc-epitopes to other *Plasmodium* proteins, such as *P. knowlesi* SBP1 and *P. knowlesi* KAHRP2, for which Myc-tags were detectable by immunoelectron microscopy; indicating that this linker sequence was at least able to expose fused Myc-epitope to be recognized by the anti-Myc antibody^16,17^. However, the molecular mechanism to translocate plasmodial molecules to the surface of the iRBC is not well understood, thus any optimized linker sequence has not yet been reported for this process.

Secondly, other alterations might be required for the higher binding; for example, we found that the transcript levels of several *P. knowlesi* ORFs encoding proteins exported to the iRBC cytosol were consistently changed in addition to *sica-huvec* (Tables 1, 2; Tables S2 and S3), which might contribute to the increased cytoadherence activity of the naturally selected lines. We consider the latter scenario is likely, but further investigation is required to clarify this point.

To understand the biological significance of the cytoadhesion, it is important to identify both the parasite ligand and the host receptor. Multiple host vascular receptors have been identified for *P. falciparum* PfEMP1, such as CD36, ICAM1, VCAM1, and chondroitin sulfate A^18^. In the case of *P. vivax*, Bernabeu et al. demonstrated that *P. falciparum* expressing *P. vivax* iRBC surface protein VIR14 bound to ICAM1 expressed on CHO cells^15^; and Chotivanich et al. showed that *P. vivax*-iRBCs collected from patients bound to immobilized glycosaminoglycans, chondroitin sulfate A, and hyaluronic acid^19^. Regarding *P. knowlesi*, iRBCs collected from patients were shown to bind to recombinant human ICAM1 and VCAM^20^. Lee et al. recently reported cytoadhesion of RBCs infected with *P. knowlesi* A1-H.1 strain^21^ to human endothelial cells (human cerebral microvascular endothelial cells, human pulmonary microvascular endothelial cells, or human renal glomerular endothelial cells) stimulated with *P. knowlesi* culture supernatant, and which was inhibited by antibodies against chondroitin sulfate proteoglycan 4 (CSPG4), ICAM1, VCAM1, PECAM1, P-selectin, or CD36^22^. Thus, these molecules are receptor candidates of these cells for cytoadhesion of *P. knowlesi*-iRBCs. As for HUVECs, comprehensive proteomics analysis revealed that of the above mentioned 6 molecules the highest expressions observed were for PECAM1 (Peptide spectrum match (PSM) = 431), followed by ICAM1 (PSM = 59), and low expression of VCAM1 (PSM = 0.21); whereas CSPG4, P-selectin, and CD36 were not detected^23^. It would be of interest to examine if the first three molecules are receptors of SICA-HUVEC. Identification of the host receptor of SICA-HUVEC would significantly increase our understanding of cytoadhesion in the pathogenesis of *knowlesi* malaria. It is also of interest to determine if the identified SICA-HUVEC has a role in the sequestration of intraerythrocytic parasites in humans.

## Materials and methods

### *P. knowlesi* culture

A *P. knowlesi* H-DMU culture-adapted parasite line was established previously^16^. Parasites were cultured in vitro with rhesus monkey RBCs in RPMI-1640 medium-based complete culture medium (CM) containing 0.5% AlbuMAX II (Invitrogen, Carlsbad, CA, USA), 200 nM hypoxanthine (Sigma Aldrich, St. Louis, MO, USA), 0.23% sodium bicarbonate, and 20 μL/mL gentamicin (Invitrogen). All monkeys used as blood donors were bred and raised in Japan at animal facilities in a malaria-free environment, and housed in individual cages (cage size: 650 [W] × 750 [D] × 820 [H] mm) in a controlled environment (22 to 25 °C, 30–60% humidity, and a 12-h light/dark cycle). They were fed with a diet standard for old-world monkeys, supplemented with fresh fruits, and water was given ad libitum. The investigators adhered to the Guidelines for the Use of Experimental Animals authorized by the Japanese Association for Laboratory Animal Science. The protocol was approved by the Committee on the Ethics of Animal Experiments of the Dokkyo Medical University (Permit Number: 0656).

To enrich schizont stage parasites, *P. knowlesi* cultures were used having more than 1% parasitemia of schizonts. The iRBCs were suspended in 5 mL of incomplete culture medium (ICM), which does not contain AlbuMax II, sodium bicarbonate, and gentamicin; then layered above a 50% Nycodenz solution (1.077 g/mL) and centrifuged at 900×*g* for 12 min at 20 °C^17^. The schizont-enriched layer was then collected, and the cells were washed twice with ICM. The schizont-iRBCs were confirmed by Giemsa-staining of blood smears.

### Binding assay of iRBCs and panning selection using human umbilical vein endothelial cells (HUVECs)

HUVECs (C-12203; PromoCell, Heidelberg, Germany) were cultured in 6-well plates coated with polystyrene (AGC Techno Glass, Shizuoka, Japan). A trypsin detachment kit (C-41200; PromoCell) was used to harvest and subculture HUVEC via passaging. An endothelial cell growth medium kit (C-22110; PromoCell) was used to maintain HUVEC cultures.

For binding assays, HUVECs were seeded and grown to high confluency on the bottom of poly-l-lysine-coated 6-well plates where poly-l-lysine-coated coverslips (13 mm ø, thickness 0.13–0.17 mm; Matsunami Glass, Osaka, Japan) were placed. Before use HUVECs were rinsed 3 times with 3 mL of pre-warmed Basal Medium (BM; C-22210; PromoCell). *P. knowlesi* parasites were cultured at a 5 mL scale until the parasitemia of trophozoite and schizont stages became more than 3%, washed with 5 mL of pre-warmed BM, adjusted for parasitemia to approximately 3.5%, resuspended in 900 μL of BM (final concentration of iRBCs was 17–19%), and then placed on HUVECs in triplicate wells for each sample (300 μL/well). The cells were incubated at 37 °C for 75 min with a 5% O_2_, 5% CO_2_, and 90% N_2_ gas mixture, with gentle shaking to suspend the iRBCs at 30 and 60 min during the incubation. After incubation, coverslips with HUVECs covered with RBCs were gently removed, washed with BM, fixed with 1% glutaraldehyde at RT for 30 min, and then stained with Giemsa's solution. The images were observed by light microscopy and digitally captured, then the numbers of iRBCs per 100 HUVECs were counted. The difference between the test and control groups was examined by one-way ANOVA followed by post-hoc Tukey's multiple comparison test or two-tailed Student's *t*-test. We used HUVECs passaged less than 10 times because it was reported that HUVECs passaged more than 15 times begin to lose their cell spreading activity^24^.

For the panning selection, the iRBCs remaining on HUVECs after removing the coverslips were washed 5 times with 3 mL of BM, and then 3 mL of *P. knowlesi* CM containing 2% uninfected rhesus monkey RBCs was added. The parasites in the 6-well plates were incubated with the above gas mixture overnight at 37 °C, then RBCs infected with newly invaded parasites were recovered and cultured in new flasks in 5 mL of CM containing approximately 2% rhesus monkey RBCs.

### RNA sequencing (RNA-seq)

Total RNAs were prepared using the TRIzol reagent. For exp-1, libraries were generated using a TruSeq Stranded mRNA Sample Preparation Kit (Illumina, San Diego, CA, USA), and then 150 bp paired-end reads were sequenced on a HiSeq2500 (Illumina). For exp-2, libraries were constructed using a Next Ultra RNA Library Prep Kit for Illumina (New England Bio Labs, Ipswich, MA, USA) and 150 bp paired-end reads sequenced on a Novaseq 6000 (Illumina). The reads obtained by RNA-seq were mapped by TopHat (version 2.1.1) to the *P. knowlesi* H strain genome sequence acquired from PlasmoDB (release-31). Aligned reads per gene were counted by HTseq (version 0.6.1), and the ORFs for which the average FPKM of all data was less than 1 were excluded from further analysis. Differentially expressed genes were identified by DESeq2 (version 1.30.1) with their *q* values, which are adjusted *P* values obtained by considering the false discovery rate. Volcano plots and correlation plots were generated using Microsoft Excel, and Spearman’s rank correlation coefficient (ρ), and significances were calculated using GraphPad Prism (version 7).

### cDNA synthesis, RT-PCR, and sequencing

Extracted RNA was treated with DNase I (Promega, Madison, WI, USA) and further purified using an SV Total RNA Isolation System kit (Promega). cDNA was synthesized using SuperScript III (Invitrogen, Carlsbad, CA, USA) following the manufacturer’s instructions. PCR was done using KOD-Plus-Neo (Toyobo, Osaka, Japan) with primer sets SICA-HUVEC.F1 and SICA-HUVEC.R1, SICA-HUVEC.F2 and SICA-HUVEC.R2, SICA-HUVEC.F3 and SICA-HUVEC.R3, SICA-HUVEC.F4 and SICA-HUVEC.R4, SICA-HUVEC.F5 and SICA-HUVEC.R5, SICA-HUVEC.F6 and SICA-HUVEC.R6, or SICA-HUVEC.F7 and SICA-HUVEC.R7 (Table S1). Amplified PCR products were directly sequenced or purified using NucleoSpin^®^ Gel and PCR Clean-up (Takara Bio, Kusatsu, Japan), ligated with pGEM^®^-T Easy plasmid (Promega), and 2 clones were sequenced using M13F primer, M13R primer, and primers designed for sequencing (Table S1). Obtained sequences were assembled and deposited to the DDBJ database under the accession number LC663824.

### Plasmid construction

Seven DNA fragments of *sica-huvec* in pGEM-T Easy plasmids, described above, were used as templates to make a single DNA fragment containing the full length ORF of *sica-huvec* by PCR using KOD-Plus-Neo (Toyobo). At the final step, PrimeSTAR DNA polymerase (Takara Bio) was used with primer sets SICA-HUVEC_Inf.F and SICA-HUVEC_Inf.R (Table S1). The purified PCR product was ligated between the BamHI and KpnI sites in a pB1/3_2Myc plasmid^16^ using an In-Fusion^®^ HD Cloning Kit (Takara Bio) to yield pB1/3_SICA-HUVEC-2Myc. BP recombination reaction between pB1/3_SICA-HUVEC-2Myc and pDONR-P1P3 was performed to obtain the entry clones pENTR1/3_SICA-HUVEC-2Myc. pENT1/3-mCherry1 was generated using PCR-amplified product containing an mCherry ORF from the pmCherry-1 plasmid (Takara Bio) with primers B1-A6-BS-hGFP-Fw and B3-S(St)Xol-hGFP-Rv (Table S1) and pDONR-P1P3. pDONR-P1P3 was constructed from pDONR™ 201 (Thermo Fisher Scientific, Waltham, MA, USA) by modifying the *att*P2 sequence to an *att*P3 sequence as described^25^. Then the pENTR1/3-based plasmids were subjected to a Gateway Multisite LR recombination reaction with pENTR4/1_PfCRT5′ and pCHD43(II)^26,27^ to yield pCHD-SICA-HUVEC-Myc and pCHD-mCherry, respectively. The plasmids pENTR4/1_PfCRT5′ and pCHDR-3/4 (origin of pCHD43(II)) were gifts from G. McFadden (University of Melbourne, Australia).

### Transgenic parasites

Synchronized *P. knowlesi* schizonts were transfected using Amaxa Nucleofector 4D (Lonza, Basel, Switzerland) and a P3 Primary cell 4D Nucleofector X Kit L (Lonza). Approximately 20 μg of plasmid DNA in double-distilled H_2_O was added to 100 μL of P3 primary cell solution. Approximately 5 × 10^7^ purified schizonts were resuspended in the DNA plus P3 primary cell solution, transferred into cuvettes (Lonza), and electroporated using program FP158. The cuvettes were immediately put on ice, then the electroporated cells were transferred to tubes containing 500 μL of pre-warmed CM with 20% rhesus monkey RBCs. The mixture was incubated on a thermo shaker (Thermomixer Comfort; Eppendorf, Hamburg, Germany) at 37 °C at 1000 rpm for 2 h, then transferred to a tissue culture flask containing 4.5 mL of CM pre-warmed to 37 °C to a final hematocrit of 2%. WR99210 was supplied in culture medium to a final concentration of 1.25 nM at 24 h post-transfection. The drug concentration was increased two-fold once drug-resistant parasites appeared, and the transgenic parasites were ultimately maintained with 10 nM WR99210.

### SDS-PAGE and Western blot

RBCs infected with unsynchronized parasites were incubated with 0.15% saponin in PBS containing a protease inhibitor (PI) cocktail (cOmplete™ ULTRA mini EASYpack; Roche, Basel, Switzerland) (PBS-PI) at 4 °C for 3 min, washed once with PBS-PI, then frozen at − 80 °C until use. The water-soluble fraction (FT fraction) was obtained by freeze-thawing (FT) the pellets in PBS-PI 3 times between 4 °C and − 80 °C. The remaining pellets were washed 3 times with PBS-PI, then incubated with 1% non-ionic detergent Triton X-100 (Tx; Calbiochem, San Diego, CA, USA) on ice for 30 min to obtain a Tx fraction. Tx-insoluble pellets were washed 3 times with PBS-PI containing Tx, then incubated with 2% sodium dodecyl sulfate (SDS; Nacalai Tesque, Kyoto, Japan) in PBS-PI at room temperature (RT) for 30 min to obtain an SDS fraction.

Proteins were separated by 5–20% gradient SDS-PAGE (ATTO, Tokyo, Japan), transferred to PVDF membrane (Merck Millipore, Burlington, Germany), and immunostained with mouse anti-Myc monoclonal antibody (1:500–1:1000; 9B11; Cell Signaling Technology, Danvers, MA, USA) at RT for 1 h, followed by incubation with HRP-conjugated anti-mouse IgG (1:10,000; Promega) at RT for 1 h. Bands were visualized using Immobilon Western Chemiluminescent HRP substrate (Merck Millipore) and images were taken by a multipurpose charge-coupled-device (CCD) camera system (ImageQuant LAS 4000 EPUB mini system; GE Healthcare, Chicago, IL, USA) with Multi Gauge software, then the intensity was adjusted using a graphics editor (Photoshop 23; Adobe Inc., San Jose, CA).

### Immunofluorescence microscopy

Indirect immunofluorescence assays (IFA) were performed for thin layer blood films on glass slides, which were prepared from mixed stage cultures with 3–10% parasitemia, air-dried at RT, then stored at − 80 °C until use. Retrieved blood films were thawed at RT in a desiccator with silica gel beads and fixed at RT for 15 min using 4% paraformaldehyde (Nacalai Tesque) and 0.075% glutaraldehyde (Nacalai Tesque) in PBS. The reaction was neutralized with 50 mM glycine (Fujifilm Wako Chemicals, Osaka, Japan) in PBS for 15 min, then blocked with 10% normal goat serum (Invitrogen) in PBS at RT for 60 min. Blood films were immunostained with mouse anti-Myc monoclonal antibody (1:500) or rabbit anti-mCherry polyclonal antibody (1:500; ab167453, Abcam, Cambridge, UK) and incubated at RT for 60 min. After washing 3 times with PBS, the blood films were incubated with a solution containing Alexa Fluor^®^ 488-conjugated goat anti-mouse IgG antibody (1:500; Invitrogen) or Alexa Fluor^®^ 594-conjugated goat anti-rabbit IgG antibody (1:500; Invitrogen) at RT for 30 min. Normal mouse or rabbit IgG (Merck Millipore) were used as negative controls. Nuclei were stained with 4′,6-diamidino-2-phenylindole (DAPI; 1:500; Invitrogen). ProLong^®^ Gold antifade reagent (Invitrogen) was used as a mounting solution. Signals were visualized using a fluorescence microscope (Axio Imager Z2; Carl Zeiss, Oberkochen, Germany) equipped with a 100 ×/1.4 oil immersion lens. Acquired images were processed and analyzed using ZEN 3.0 blue edit software (Carl Zeiss). For some blood films, immunostained samples were Giemsa-stained as described^16^.

Double immunostainings of the blood films were performed similar to above using mouse anti-Myc monoclonal antibody, rat anti-human CD235a (glycophorin A) antibody (1:500; YTH 89.1, Bio-Rad Laboratories, Hercules, CA, USA), and rabbit anti-mCherry polyclonal antibody as primary antibodies; and Alexa Fluor^®^ 488-conjugated goat anti-mouse, Alexa Fluor^®^ 488-conjugated goat anti-rabbit, and Alexa Fluor^®^ 594-conjugated goat anti-rat (1:500; Invitrogen) IgG antibodies as secondary antibodies. Normal mouse, rabbit, and rat IgG (Merck Millipore) were used as negative controls.

### Quantitative RT-PCR (qRT-PCR)

To evaluate the transcription levels of SICA-HUVEC, qRT-PCR was performed using Power SYBR^®^ Green PCR Master Mix (Thermo Fisher Scientific) and a 7500 Real-Time PCR system (Thermo Fisher Scientific). Two primer sets were used for *sica-huvec*: SICA-HUVEC.rt-F1 and SICA-HUVEC.rt-R1, and SICA-HUVEC.rt-F2 and SICA-HUVEC.rt-R2. As a control, PkMet-tRNA.rt-F and PkMet-tRNA.rt-R were used for *methionine tRNA ligase* (Table S1).

### Trypsin cleavage assay

RBCs infected with *P. knowlesi* expressing SICA-HUVEC-Myc were incubated at ~ 30% hematocrit and parasitemia of 7.6% (trophozoites + schizonts) with 500 µg/mL trypsin and 0.05% EDTA in Hanks' balanced salt solution (Cat. 25300054; Thermo Fisher Scientific) at 37 °C for 5 min using a thermo shaker at 1000 rpm (ThermoMixer Comfort). As a control, iRBCs were also incubated in Hanks' balanced salt solution without Trypsin-EDTA. iRBCs were then incubated with 1 mg/mL soybean trypsin inhibitor (Nacalai Tesque) in ICM at RT for 15 min. Proteins were sequentially extracted from iRBCs as described above.

### Ethical standards

This study was conducted in accordance with ARRIVE guidelines.

## Supplementary Information

Supplementary Information.

## Data Availability

Nucleotide sequence data were deposited to the DDBJ database under the accession number LC663824. RNA-seq datasets are available from DDBJ (BioProject Accession Number PRJDB12907; https://www.ncbi.nlm.nih.gov/bioproject/).
