# Supplementary material for: SICA-mediated cytoadhesion of Plasmodium knowlesi-infected red blood cells to human umbilical vein endothelial cells
Source: Sci Rep. 2022 Sep 2;12:14942. doi: 10.1038/s41598-022-19199-0 (PMC9440145; doi:10.1038/s41598-022-19199-0)
Supplement: Supplementary file 1 — Supplementary Information. [file 41598_2022_19199_MOESM1_ESM.pdf]

Table S1. Oligonucleotides used in this study

name	sequence (5'->3')	comment
SICA-HUVEC.F1	TATATATAGAGGAATATTTGCAAAGAAG	To amplify *P. knowlesi sica-huvec*
SICA-HUVEC.R1	GGCGCTGTCCACTATTCCTAGG	
SICA-HUVEC.F2	GGTACCCAAGTGCGCATAATCAAC	
SICA-HUVEC.R2	GACTTGGTCACAGAGTTCTGTTAC	
SICA-HUVEC.F3	GGAAAAATTGAAAGAAATTGTCAAAGC	
SICA-HUVEC.R3	CACTGTTTAACATTAAGCAGGAAGCG	
SICA-HUVEC.F4	TTCAGCGGAACTACACCACGAAG	
SICA-HUVEC.R4	TGAACATGTGTTAACTTCTCCTTTAC	
SICA-HUVEC.F5	GAATGAAGAAATCCTTACTACTTGC	
SICA-HUVEC.R5	GAAGTTTCTTTAATTGCTCATCAACG	
SICA-HUVEC.F6	AAACAATACCACAAAAATAAGCATAATG	
SICA-HUVEC.R6	TTGCAATTGTTTTGCATACTCTTTAAG	
SICA-HUVEC.F7	TAAAACACATTTATACTCATGATAATGG	
SICA-HUVEC.R7	ACCCGAAACCCTAAACACTGAACCC	
SICA-HUVECseqF1	TTCACGGGTGTGGAGGCCCAGGA	To sequence *P. knowlesi sica-huvec*
SICA-HUVECseqR1	CCACTCGTGTTCTTCAATGGTTGTT	
SICA-HUVECseqF2	TTGGGCAGAGAATGATGGAGCAGTG	
SICA-HUVECseqR2	CTTATACAGATGTTCTAAGCCGGCA	
SICA-HUVECseqF3	GCCATTGCAGATAAACTACAAGACAAC	
SICA-HUVECseqR3	CCTTAATTTTCGTCATTTCTATGTTTTCGC	
SICA-HUVECseqF4	AGCATCTCGAGTTCAAGCTGAAGCA	
SICA-HUVECseqR4	TGCCAGTGGCACTGACATCATCCAT	
SICA-HUVECseqF5	ATCGCGGCAGGACTACAACACATTA	
SICA-HUVECseqR5	GCAGAGGTCAAGCACGAATTATTATTAA	
SICA-HUVECseqF6	GATAATAGTAAAACCATTATGAATGGAGGAAC	
SICA-HUVECseqR6	CTTCTAGTGTTTTGTCCATGGTAGG	
SICA-HUVECseqF7	TCCCATCCTTCTTACGGATCTCCTT	
SICA-HUVECseqR7	GGTCCCCCTTTACCAAGAGGACCAA	
SICA-HUVEC_Inf.F	AGGTCGACTCTAGA**GGATCC**acaacaATGTCGACAGTAGGAAATGGTGC	To amplify the full length of *sica-huvec* ORF. BamHI and KpnI sites are bolded
SICA-HUVEC_Inf.R	CCGTCGAGCAGCTC**GGTACC**AACCAGTAACCCGGAACCTAAACTTGG	
B1-A6-BS-hGFP-Fw	GGGGCAAGTTTGTACAAAAAAGCAGAAAAAAATGGGATCCACTAGTGTGAGCAAGGGCGAGGAG	To amplify mCherry ORF
B3-S(St)Xol-hGFP-Rv	GGGGCAACTTTGTATAATAAAGTTGCCCGGGTTACTCGAGCTTGTACAGCTCGTCC	
SICA-HUVEC.rt-F1	CAGTAGGAAATGGTGCCAGCGGAGG	For qPCR of *P. knowlesi sica-huvec*
SICA-HUVEC.rt-R1	CCATAATGTCCTTCCCACTGTTCAGA	
SICA-HUVECrt-F2	GTGGACAGCGCCAAGGTTGAGAATA	
SICA-HUVECrt-R2	CGCCCACTTTGCTCGGGTCCACGGT	
PkMet-tRNA.rt-F	AAAAATGTCCAAATCGTTAGGCAATGTG	For qPCR of *P. knowlesi methionine tRNA ligase*
PkMet-tRNA.rt-R	TCGGCTAAGTCGGAGTTACACATATC	

Table S2. *P. knowlesi* ORFs predicted in PlasmoDB with increased transcript expression after panning selection

	ID	annotation	Fragments per kilobase of transcript per million reads mapped (FPKM)							Log2 fold change after panning				*P* value	*q* value
			exp-1				exp-2			exp-1		exp-2			
			pre-1	pre-2	6th pan	8th pan	pre-3	well-1	well-2	6th pan	8th pan	well-1	well-2		
4 hours (ring form early trophozoite)	PKNH_0814300	SICAvar, type I (fragment)	4.47	4.09	1183.34	690.22	2.68	52.59	65.09	8.05	7.27	4.29	4.60	1.34×10^{-6}	8.93×10^{-4}
	PKNH_0814200	SICAvar, type II (fragment)	2.25	1.14	526.93	327.52	3.17	22.62	26.92	7.87	7.19	2.84	3.09	6.88×10^{-6}	4.01×10^{-3}
	PKNH_0322100	SICAvar, type I (fragment)	4.42	8.36	147.92	92.07	11.01	19.60	14.90	5.06	4.38	0.83	0.44	0.002	> 0.5
	PKNH_1214500	PIR protein	1.43	0.24	7.56	8.13	4.23	7.26	19.20	2.40	2.51	0.78	2.18	0.002	> 0.5
	PKNH_1247200	SICAvar, type I (fragment)	0.26	0.70	5.13	4.22	0.30	1.03	0.98	4.30	4.02	1.78	1.70	0.013	> 0.5
8 hours (late trophozoite)	PKNH_0814300	SICAvar, type I (fragment)	3.07	4.69	1380.68	895.07	4.11	67.30	66.57	8.81	8.19	4.03	4.02	3.20×10^{-7}	8.26×10^{-4}
	PKNH_0814200	SICAvar, type II (fragment)	2.44	2.67	637.39	442.40	3.39	29.68	32.75	8.03	7.51	3.13	3.27	2.63×10^{-6}	4.53×10^{-3}
	PKNH_0322100	SICAvar, type I (fragment)	7.45	9.33	171.11	114.40	8.42	17.81	15.29	4.52	3.94	1.08	0.86	2.16×10^{-4}	> 0.1
24 hours (schizont)	PKNH_0814300	SICAvar, type I (fragment)	1.86	1.14	493.57	398.36	3.48	52.43	45.23	8.05	7.74	3.91	3.70	8.61×10^{-13}	4.59×10^{-9}
	PKNH_1033000	SICAvar, type II	1.36	1.11	1.88	3.69	24.91	262.98	472.15	0.47	1.44	3.40	4.24	0.004	> 0.5
	PKNH_0814200	SICAvar, type II (fragment)	0.85	1.81	230.53	156.26	3.91	26.45	18.23	8.09	7.53	2.76	2.22	2.13×10^{-9}	5.67×10^{-6}
	PKNH_0946500	SICAvar, type II	0.03	0.03	0.13	0.25	5.35	31.76	73.35	1.94	2.83	2.57	3.78	0.037	> 0.5
	PKNH_1312400	conserved protein in *P. knowlesi*	0.00	0.12	0.00	0.12	0.81	6.09	12.59	∞	∞	2.91	3.96	0.003	> 0.5
	PKNH_1100100	conserved protein in *P. knowlesi*	0.00	0.05	0.12	0.23	0.58	3.13	7.40	∞	∞	2.42	3.66	0.002	> 0.5
	PKNH_1149600	conserved protein in *P. knowlesi*	2.18	0.00	4.02	1.23	2.71	26.36	56.08	0.88	–0.83	3.28	4.37	0.002	> 0.5
	PKNH_1463900	conserved protein in *P. knowlesi*	0.00	0.24	0.00	0.49	0.00	2.33	0.64	∞	∞	∞	∞	0.048	> 0.5
	PKNH_0727500	PIR protein	0.00	0.00	1.32	1.86	0.26	1.23	0.10	∞	∞	2.27	–1.34	0.044	> 0.5
	PKNH_0200300	SICAvar, type II	2.09	3.03	7.89	14.88	46.21	161.06	322.70	1.92	2.83	1.80	2.80	0.002	> 0.5
	PKNH_0300400	SICAvar, type II	0.34	0.13	2.21	5.38	14.61	46.88	80.31	2.71	4.00	1.68	2.46	0.049	> 0.5
	PKNH_0300200	SICAvar, type II	0.19	0.56	0.48	0.39	1.91	6.34	13.39	1.35	1.02	1.73	2.81	0.008	> 0.5
	PKNH_0321900	PIR protein	0.04	0.11	0.05	0.12	0.82	2.61	4.36	0.35	1.61	1.68	2.42	0.017	> 0.5
	PKNH_1247200	SICAvar, type I (fragment)	0.10	0.10	2.99	2.38	0.23	0.90	0.46	4.85	4.51	1.94	0.99	0.001	> 0.1
	PKNH_1021100	SICAvar, type I (fragment)	0.66	2.27	11.00	11.44	31.18	188.16	31.46	4.05	4.11	2.59	0.01	0.019	> 0.5
	PKNH_1263500	Ca–dependent protein kinase 5	16.43	3.12	6.59	3.58	19.28	74.64	198.12	–1.32	–2.20	1.95	3.36	0.008	> 0.5
	PKNH_0100400	*Plasmodium* exported protein	44.38	17.97	29.37	17.58	32.91	153.69	452.96	–0.60	–1.34	2.22	3.78	0.004	> 0.5
	PKNH_1247700	PHIST	5.33	1.93	4.06	1.90	2.31	15.33	46.26	–0.39	–1.49	2.73	4.33	0.007	> 0.5
	PKNH_0400300	*Plasmodium* exported protein	2.67	1.05	1.23	1.73	2.39	9.89	26.90	–1.12	–0.62	2.05	3.49	0.004	> 0.5
	PKNH_1325900	*Plasmodium* exported protein	4.21	1.90	3.39	0.86	1.06	10.75	36.40	–0.31	–2.30	3.34	5.10	0.015	> 0.5

All ORFs whose average fold change values were more than or equal to 4 and *P* value < 0.05 are shown. Log2 fold changes are accompanied with heatmap colors (red > 0 and blue < 0). *P* values were obtained excluding the exp-1 6th pan data.

Table S2. *P. knowlesi* ORFs predicted in PlasmoDB with increased transcript expression after panning selection (cont')

	ID	annotation	Fragments per kilobase of transcript per million reads mapped (FPKM)							Log2 fold change after panning				*P* value	*q* value
			exp-1				exp-2			exp-1		exp-2			
			pre-1	pre-2	6th pan	8th pan	pre-3	well-1	well-2	6th pan	8th pan	well-1	well-2		
24 hours (schizont) (cont')	PKNH_0830000	SICAvar, type II	2.85	2.11	4.24	4.08	3.09	14.86	30.41	0.57	0.52	2.27	3.30	2.48×10^{-4}	> 0.1
	PKNH_0100300	SICAvar, type I (fragment)	0.00	0.03	0.08	0.33	0.47	0.86	1.42	∞	∞	0.89	1.61	0.046	> 0.5
	PKNH_1149800	SICAvar, type I (fragment)	0.00	0.04	0.29	0.85	0.33	0.54	0.66	∞	∞	0.69	0.99	0.018	> 0.5
	PKNH_0826200	conserved *Plasmodium* protein	0.13	0.13	0.59	0.67	1.18	1.92	4.93	2.16	2.34	0.70	2.06	0.019	> 0.5
	PKNH_0619000	SICAvar, type I (fragment)	0.27	0.13	0.17	0.69	0.10	1.02	1.20	–0.65	1.34	3.34	3.57	0.036	> 0.5
	PKNH_1247400	*Plasmodium* exported protein	12.09	3.98	7.28	4.00	10.45	38.44	105.75	–0.73	–1.59	1.88	3.34	0.008	> 0.5
	PKNH_1300800	tryptophan-rich antigen	1.57	0.33	0.79	0.63	0.78	4.25	9.99	–0.99	–1.32	2.44	3.68	0.015	> 0.5
	PKNH_0807800	PIR protein	2.78	2.40	13.99	15.94	9.77	31.71	29.16	2.33	2.52	1.70	1.58	3.32×10^{-5}	0.0354
	PKNH_0400500	tryptophan–rich antigen	4.91	1.42	2.75	1.33	1.53	9.21	32.52	–0.83	–1.89	2.59	4.41	0.019	> 0.5
	PKNH_0401200	PHIST	1.62	0.61	1.09	1.06	0.92	3.46	12.13	–0.58	–0.61	1.90	3.72	0.008	> 0.5
	PKNH_0715200	conserved *Plasmodium* membrane protein	1.81	2.49	1.85	10.68	25.17	35.93	85.23	0.03	2.56	0.51	1.76	0.022	> 0.5
	PKNH_1247100	SICAvar, type I (fragment)	0.00	0.18	2.37	4.34	2.22	3.60	3.62	∞	∞	0.70	0.71	0.043	> 0.5
	PKNH_1121400	conserved *Plasmodium* protein	0.19	0.54	0.39	0.56	1.15	2.39	5.81	1.09	1.61	1.06	2.33	0.017	> 0.5
	PKNH_1325400	SICAvar, type II	1.51	1.33	8.46	20.38	35.00	59.49	79.51	2.48	3.75	0.77	1.18	0.029	> 0.5
	PKNH_1100200	SICAvar, type II	1.46	1.50	7.24	19.68	30.78	48.13	73.34	2.32	3.76	0.64	1.25	0.025	> 0.5
	PKNH_1248100	*Plasmodium* exported protein	13.75	6.34	8.26	3.44	8.20	31.75	104.04	–0.74	–2.00	1.95	3.66	0.019	> 0.5
	PKNH_1149700	*Plasmodium* exported protein	3.57	2.08	13.40	10.31	13.58	34.99	40.00	1.91	1.53	1.37	1.56	0.002	> 0.5
	PKNH_1401100	*Plasmodium* exported protein	1.78	0.53	1.29	0.79	0.98	3.34	11.69	–0.47	–1.18	1.76	3.57	0.025	> 0.5
	PKNH_0600800	PHISTc	1.88	0.65	1.03	0.43	0.48	3.52	11.08	–0.86	–2.13	2.87	4.53	0.027	> 0.5
	PKNH_0700200	reticulocyte binding protein	61.60	31.31	32.04	12.19	17.03	116.27	397.78	–0.94	–2.34	2.77	4.55	0.031	> 0.5
	PKNH_1248000	tryptophan–rich antigen	33.92	6.84	14.45	10.81	20.62	74.21	185.97	–1.23	–1.65	1.85	3.17	0.027	> 0.5
	PKNH_0113600	PIR protein	0.50	0.49	2.77	4.74	1.37	2.87	2.81	2.47	3.25	1.07	1.04	0.004	> 0.5
	PKNH_0300500	PHIST	0.72	0.41	0.64	0.51	0.46	1.88	4.44	–0.16	–0.49	2.04	3.28	0.010	> 0.5
	PKNH_0311900	SICAvar, type II	0.81	0.85	2.91	2.97	2.63	8.53	6.21	1.84	1.87	1.70	1.24	0.001	> 0.1
	PKNH_0312000	PIR protein	0.78	0.74	1.40	2.26	1.12	5.10	4.00	0.84	1.53	2.18	1.83	5.21×10^{-6}	9.25×10^{-3}
	PKNH_1353400	conserved *Plasmodium* protein	25.53	11.94	17.39	15.00	21.93	57.62	174.07	–0.55	–0.77	1.39	2.99	0.011	> 0.5
	PKNH_0946000	PHIST	13.79	4.09	9.33	9.42	15.23	33.97	91.52	–0.56	–0.55	1.16	2.59	0.013	> 0.5
	PKNH_1029300	conserved *Plasmodium* protein	0.08	0.22	0.58	0.92	0.56	1.63	0.94	2.94	3.61	1.53	0.73	0.022	> 0.5

All ORFs whose average fold change values were more than or equal to 4 and *P* value < 0.05 are shown. Log2 fold changes are accompanied with heatmap colors (red > 0 and blue < 0). *P* values were obtained excluding the exp-1 6th pan data.

Table S3. *P. knowlesi* ORFs predicted in PlasmoDB with decreased transcript expression after panning selection

	ID	annotation	Fragments per kilobase of transcript per million reads mapped (FPKM)							Log2 fold change after panning				*P* value	*q* value
			exp-1				exp-2			exp-1		exp-2			
			pre-1	pre-2	6th pan	8th pan	pre-3	well-1	well-2	6th pan	8th pan	well-1	well-2		
4 hours (ring form early trophozoite)	PKNH_0508300	PHIST	179.43	316.28	0.00	0.09	0.00	0.00	0.00	–∞	–10.92	NA	NA	2.66×10^{-4}	> 0.1
	PKNH_1144500	conserved protein in *P. knowlesi*	37.56	34.16	0.58	0.64	0.00	0.35	0.40	–6.01	–5.88	∞	∞	0.002	> 0.5
	PKNH_1401400	*Plasmodium* exported protein	0.72	0.00	0.00	0.00	0.00	0.00	0.00	–∞	–∞	NA	NA	0.031	> 0.5
	PKNH_1401600	*Plasmodium* exported protein	2.26	0.23	0.00	0.00	0.00	0.00	0.00	–∞	–∞	NA	NA	0.030	> 0.5
	PKNH_1144600	SICAvar, type II	127.56	91.68	2.74	1.74	5.78	3.84	4.11	–5.54	–6.19	–0.59	–0.49	5.47×10^{-10}	6.38×10^{-7}
	PKNH_0214100	SICAvar, type I (fragment)	0.13	0.21	0.00	0.00	0.09	0.00	0.04	–∞	–∞	–∞	–1.40	0.027	> 0.5
	PKNH_1401700	*Plasmodium* exported protein	91.27	56.89	7.51	10.21	11.47	8.69	9.67	–3.60	–3.16	–0.40	–0.25	1.01×10^{-5}	5.24×10^{-3}
	PKNH_0611000	conserved *Plasmodium* protein	1.46	2.70	0.52	0.23	0.62	0.46	0.23	–1.50	–2.68	–0.41	–1.40	0.030	> 0.5
	PKNH_1002800	PIR protein	30.07	21.07	8.29	5.10	2.96	2.72	4.13	–1.86	–2.56	–0.12	0.48	0.002	> 0.5
	PKNH_0727400	SICAvar, type I	34.72	49.92	14.00	9.51	16.94	8.81	8.79	–1.31	–1.87	–0.94	–0.95	1.26×10^{-6}	8.93×10^{-4}
	PKNH_1357100	*Plasmodium* exported protein	1388.47	1072.60	168.81	235.61	366.17	315.45	337.26	–3.04	–2.56	–0.22	–0.12	2.11×10^{-5}	9.84×10^{-3}
8 hours (late trophozoite)	PKNH_0508300	PHIST	172.29	161.67	0.09	0.00	0.00	0.00	0.00	–10.90	–∞	NA	NA	0.001	> 0.5
	PKNH_1144600	SICAvar, type II	166.76	160.66	3.70	1.88	8.85	2.88	4.21	–5.49	–6.47	–1.62	–1.07	1.82×10^{-14}	9.40×10^{-11}
	PKNH_1144500	conserved protein in *P. knowlesi*	58.19	54.71	1.36	1.70	1.69	0.72	0.62	–5.41	–5.10	–1.24	–1.45	3.83×10^{-5}	0.0494
	PKNH_1230900	conserved *Plasmodium* protein	1.86	0.00	0.00	0.00	0.00	0.00	0.00	–∞	–∞	NA	NA	0.044	> 0.5
	PKNH_1002800	PIR protein	48.54	37.15	9.53	12.10	5.77	2.44	2.64	–2.35	–2.00	–1.24	–1.13	0.002	> 0.5
24 hours (schizont)	PKNH_0508300	PHIST	965.81	927.60	0.00	0.31	0.00	0.00	0.00	–∞	–11.60	NA	NA	0.001	> 0.1
	PKNH_1144500	conserved protein in *P. knowlesi*	1.69	1.24	0.18	0.14	0.21	0.15	0.12	–3.23	–3.56	–0.47	–0.75	0.036	> 0.5

All ORFs whose average fold change values were less than or equal to –4 and *P* value < 0.05 are shown. Log2 fold changes are accompanied with heatmap colors (red > 0 and blue < 0). NA (not available) indicates the numerator and the denominator are both zero. *P* values were obtained excluding the exp-1 6th pan data.

Table S4. The change of the FPKM values of selected ORFs during the experiment 2 before panning (pre-3), when iRBCs bound to HUVECs (at 13th pan), and 53 days later without panning selection (53 days later).

ID	well	FPKM values		
		pre-3	at 13th pan	53 days later
**4 hours (ring form early trophozoite)**				
**PKNH_0814200***	**well-1**	**3.17**	**22.62**	**0.99**
	**well-2**	**3.17**	**26.92**	**0.76**
**PKNH_0814300***	**well-1**	**2.68**	**52.59**	**1.00**
	**well-2**	**2.68**	**65.09**	**0.68**
PKNH_1144600†	well-1	5.78	3.84	6.83
	well-2	5.78	4.11	7.02
PKNH_0727400	well-1	16.94	8.81	2.37
	well-2	16.94	8.79	2.43
PKNH_1401700	well-1	11.47	8.69	0.40
	well-2	11.47	9.67	0.39
PKNH_1357100	well-1	366.17	315.45	64.67
	well-2	366.17	337.26	67.73
**8 hours (late trophozoite)**				
**PKNH_0814200***	**well-1**	**3.39**	**29.68**	**0.83**
	**well-2**	**3.39**	**32.75**	**0.59**
**PKNH_0814300***	**well-1**	**4.11**	**67.30**	**0.57**
	**well-2**	**4.11**	**66.57**	**0.57**
PKNH_1144600†	well-1	8.85	2.88	5.10
	well-2	8.85	4.21	4.68
PKNH_1144500†	well-1	1.69	0.72	1.33
	well-2	1.69	0.62	1.06
**24 hours (schizont)**				
**PKNH_0814200***	**well-1**	**3.91**	**26.45**	**0.37**
	**well-2**	**3.91**	**18.23**	**0.44**
**PKNH_0814300***	**well-1**	**3.48**	**52.43**	**0.19**
	**well-2**	**3.48**	**45.23**	**0.14**
PKNH_0312000*	well-1	1.12	5.10	0.55
	well-2	1.12	4.00	0.84
PKNH_0807800*	well-1	9.77	31.71	0.59
	well-2	9.77	29.16	0.58

Asterisks indicate ORFs whose FPKM values significantly increased with increasing cell adhesion activity and decreased with loss of the adhesion activity. Daggers indicate ORFs whose FPKM values significantly decreased with increasing cell adhesion activity and increased with loss of the adhesion activity.

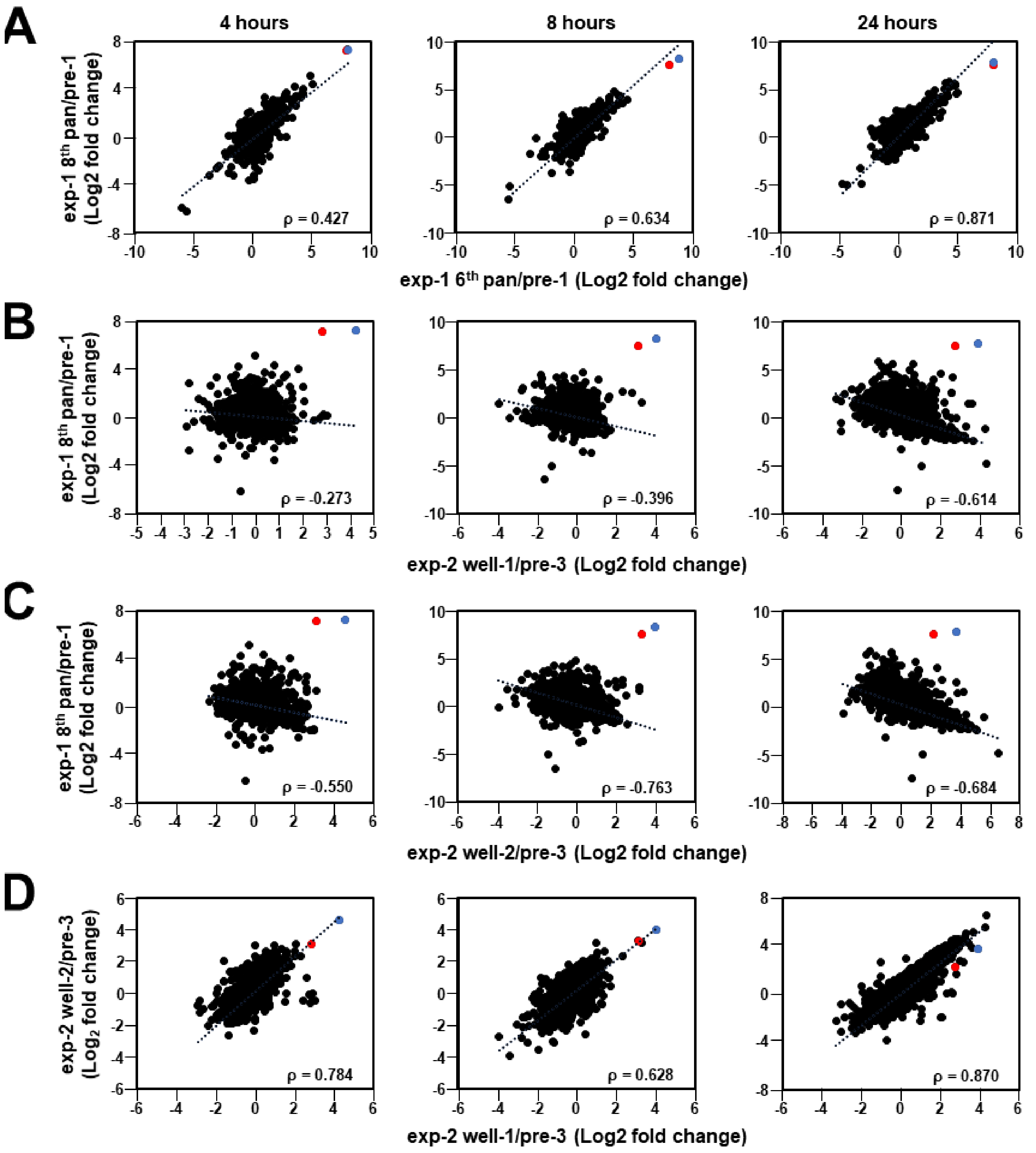

Fig. S1. Correlation plots of log2 fold changes (after panning/before panning) at different time points, 4 hours (left), 8 hours (middle), and 24 hours (right) after invasion using pre-1 samples for exp-1. PKNH_0814200 and PKNH_0814300 are highlighted in red and blue, respectively. (A) Correlation between the 6th and 8th pans of exp-1. (B) Correlation between exp-2 well-1 and exp-1 8th pan. (C) Correlation between exp-2 well-2 and exp-1 8th pan. (D) Correlation between exp-2 well-1 and well-2. Spearman's ρ values are shown.

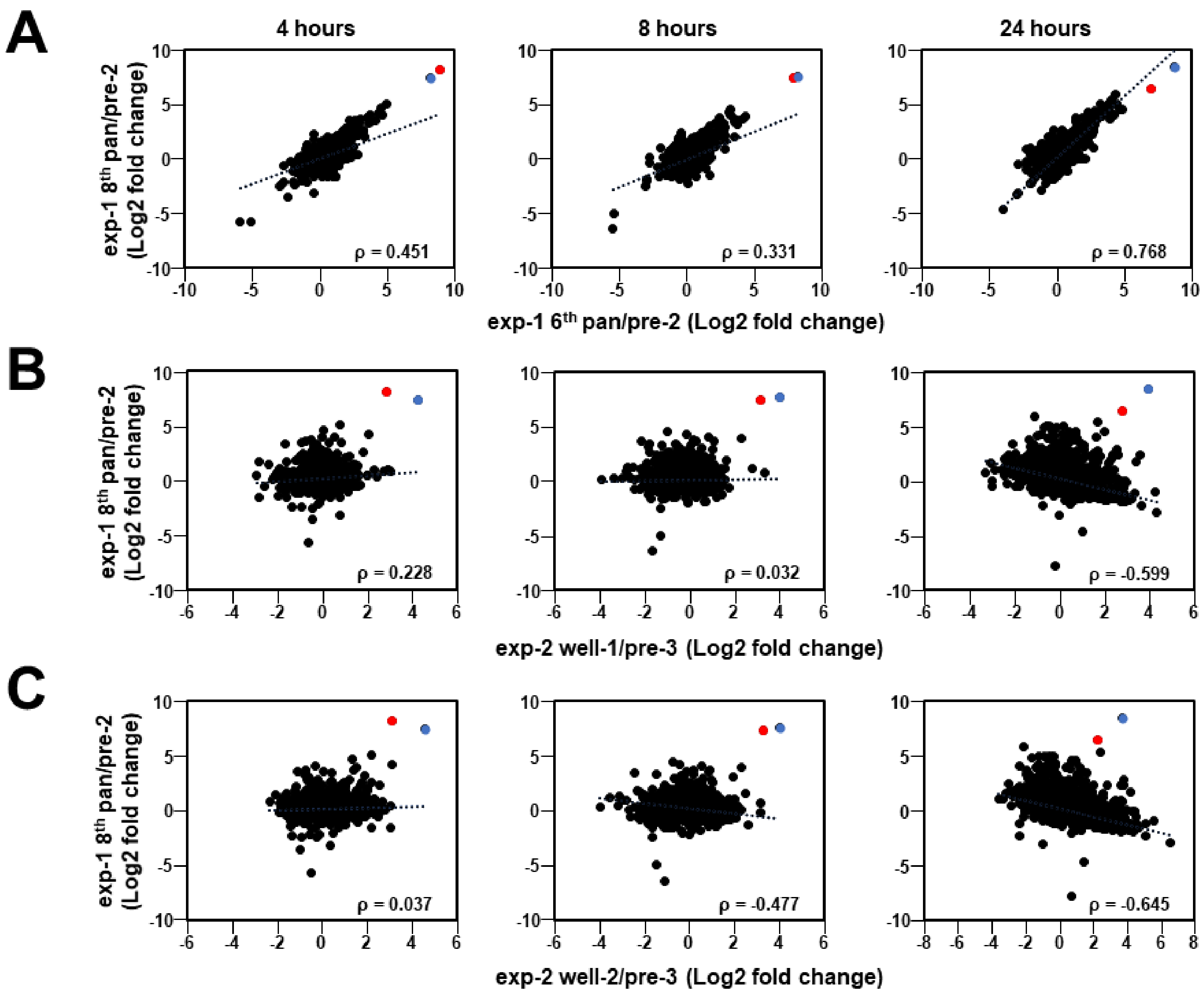

Fig. S2. Correlation plots of log2 fold changes (after panning/before panning) at different time points, 4 hour (left), 8 hour (middle), and 24 hour (right) after invasion using pre-2 samples for exp-1. PKNH_0814200 and PKNH_0814300 are highlighted in red and blue, respectively. (A) Correlation between exp-1 6th pan and 8th pan. (B) Correlation between exp-2 well-1 and exp-1 8th pan. (C) Correlation between exp-2 well-2 and exp-1 8th pan. Spearman's ρ values are shown.

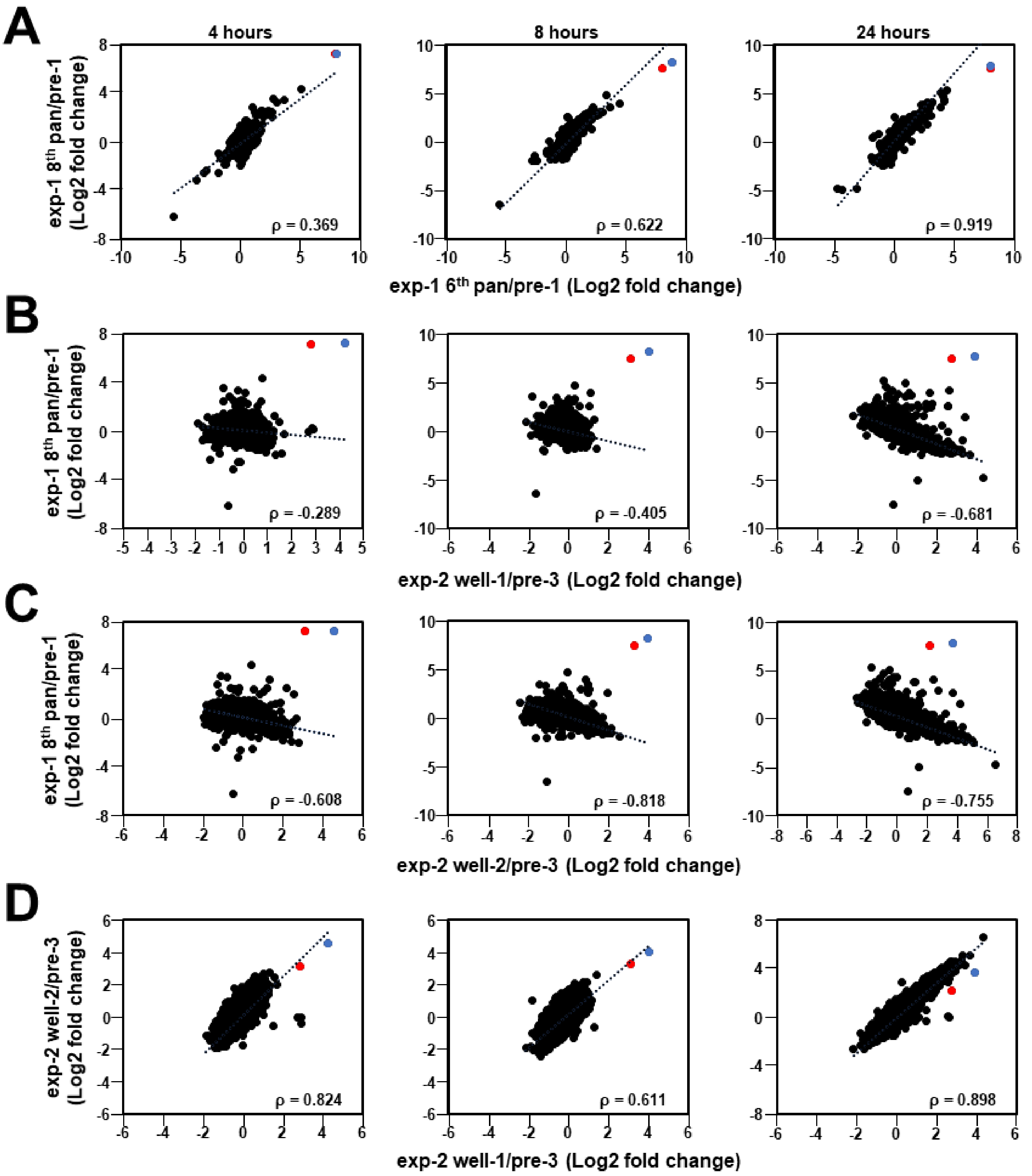

Fig. S3. Correlation plots of log2 fold changes (after panning/before panning) at different time points, 4 hours (left), 8 hours (middle), and 24 hours (right) after invasion using pre-1 samples for exp-1 (FPKM ≥ 10). PKNH_0814200 and PKNH_0814300 are highlighted in red and blue, respectively. (A) Correlation between the 6th and 8th pans of exp-1. (B) Correlation between exp-2 well-1 and exp-1 8th pan. (C) Correlation between exp-2 well-2 and exp-1 8th pan. (D) Correlation between exp-2 well-1 and well-2. Spearman's ρ values are shown. Genes with low coverage (FPKM < 10) were excluded from the analysis.

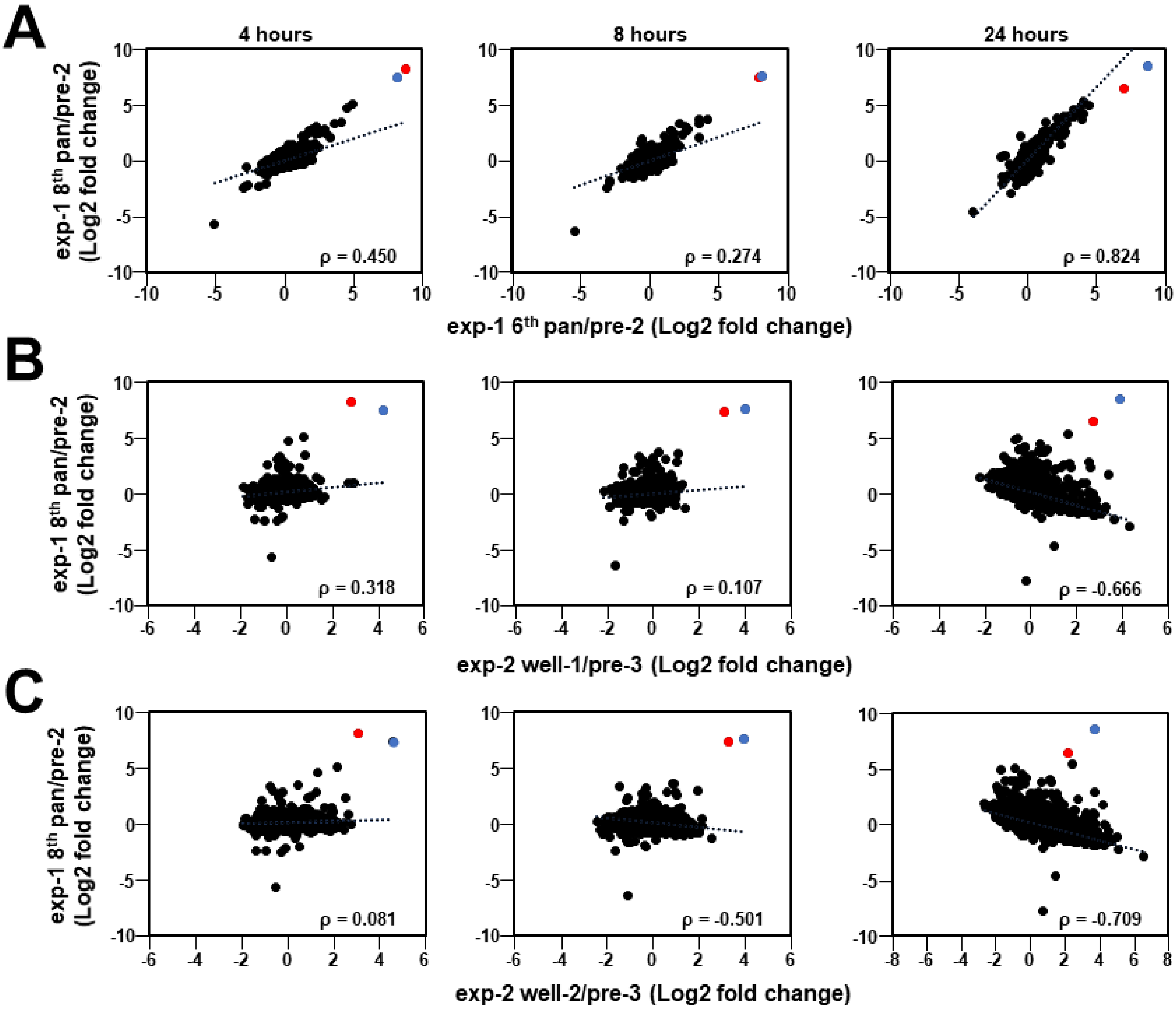

Fig. S4. Correlation plots of log2 fold changes (after panning/before panning) at different time points, 4 hour (left), 8 hour (middle), and 24 hour (right) after invasion using pre-2 samples for exp-1 (FPKM ≥ 10). PKNH_0814200 and PKNH_0814300 are highlighted in red and blue, respectively. (A) Correlation between exp-1 6th pan and 8th pan. (B) Correlation between exp-2 well-1 and exp-1 8th pan. (C) Correlation between exp-2 well-2 and exp-1 8th pan. Spearman's ρ values are shown. Genes with low coverage (FPKM < 10) were excluded from the analysis.

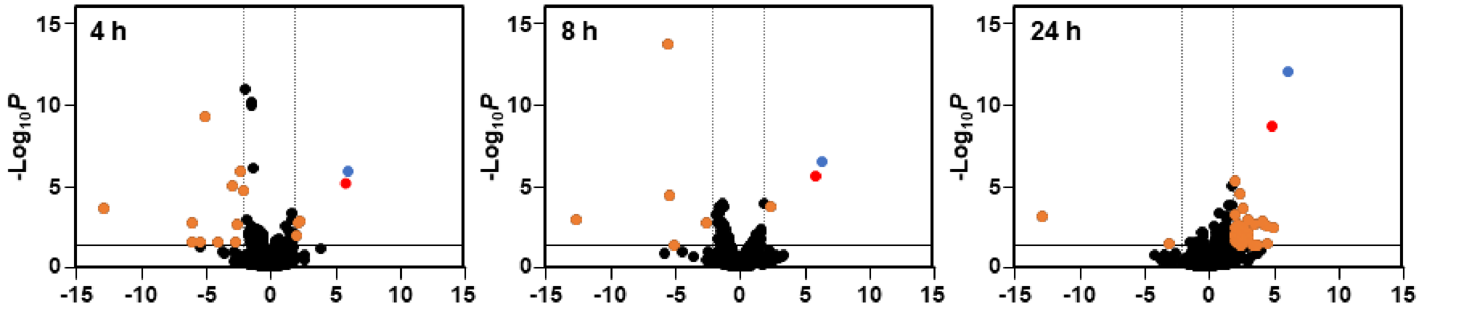

Fig. S5. RNA-seq data analysis of cytoadherent and wild-type *P. knowlesi* using all genes. Volcano plots showing log2 fold changes of transcript amounts by panning selection plotted against the $-\log_{10}$ of P value obtained by the comparison between non-binding control samples (pre-1, -2, and -3) and binding parasites samples (8th pan of exp-1, wells 1 and 2 of exp-2 13th pan) at three different time points. The dashed and solid lines indicate absolute log2 fold change = 2 and $P = 0.05$, respectively. Genes significantly differentially expressed ($P < 0.05$) and at least 4-fold changed are shown in orange dots. Genes with non-significant differences or less than 4 fold changed are shown in black dots, respectively. Gene fragments of PKNH_0814200 and PKNH_0814300 are highlighted in red and blue, respectively.

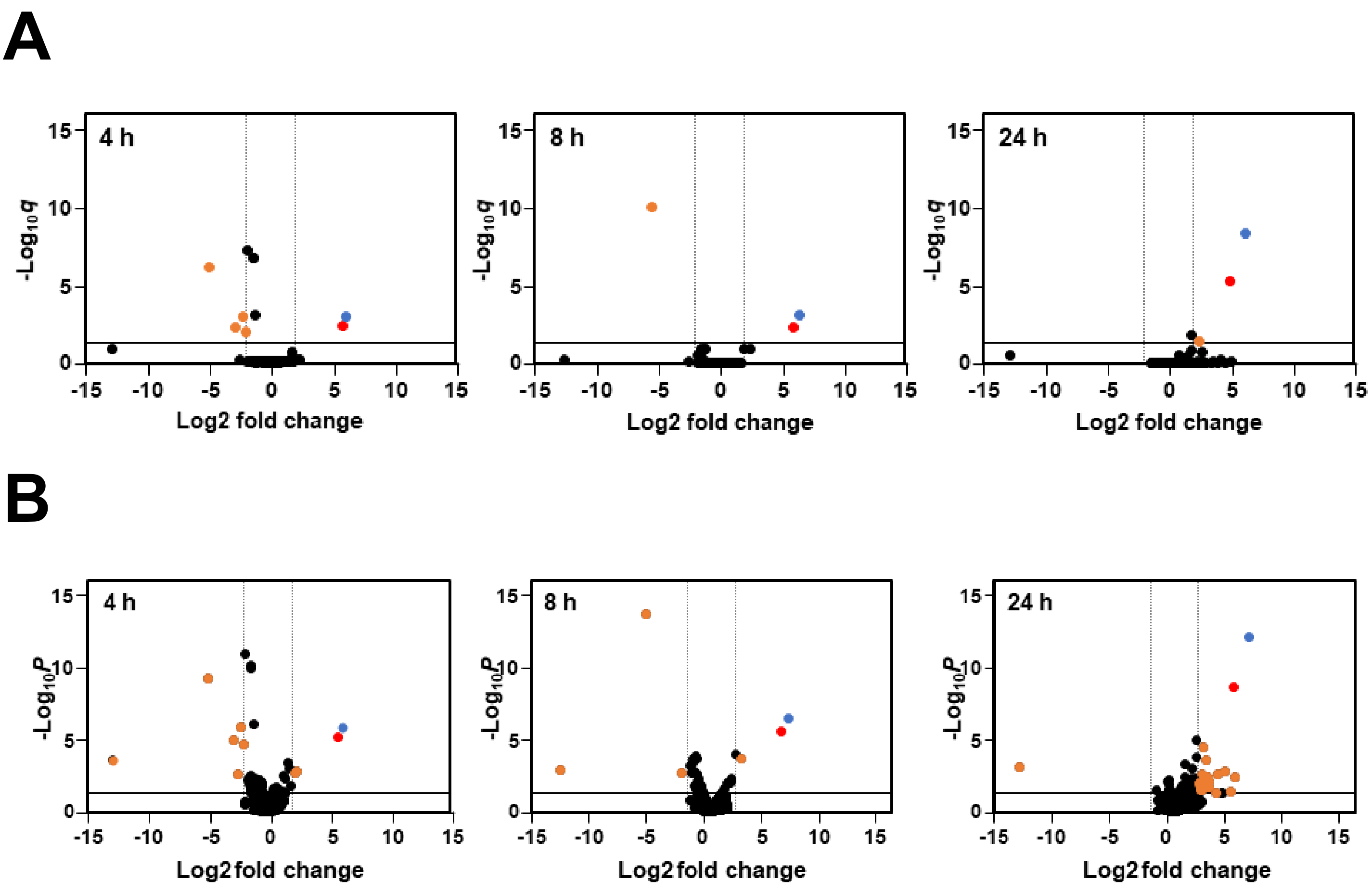

Fig. S6. RNA-seq data analysis of cytoadherent and wild-type *P. knowlesi* using genes with FPKM ≥ 10. Volcano plots showing log2 fold changes of transcript amounts by panning selection plotted against the $-\log_{10}$ of q value (A) or the $-\log_{10}$ of P value (B) obtained by the comparison between non-binding control samples (pre-1, -2, and -3) and binding parasites samples (8th pan of exp-1, wells 1 and 2 of exp-2 13th pan) at three different time points. The dashed and solid lines indicate absolute log2 fold change = 2 and $q = 0.05$ or $P = 0.05$, respectively. Genes significantly differentially expressed ($q < 0.05$ or $P < 0.05$) and at least 4-fold changed are shown in orange dots. Genes with non-significant differences or less than 4 fold changed are shown in black dots, respectively. Gene fragments of PKNH_0814200 and PKNH_0814300 are highlighted in red and blue, respectively. Genes with low coverage (FPKM < 10) were excluded from the analysis.

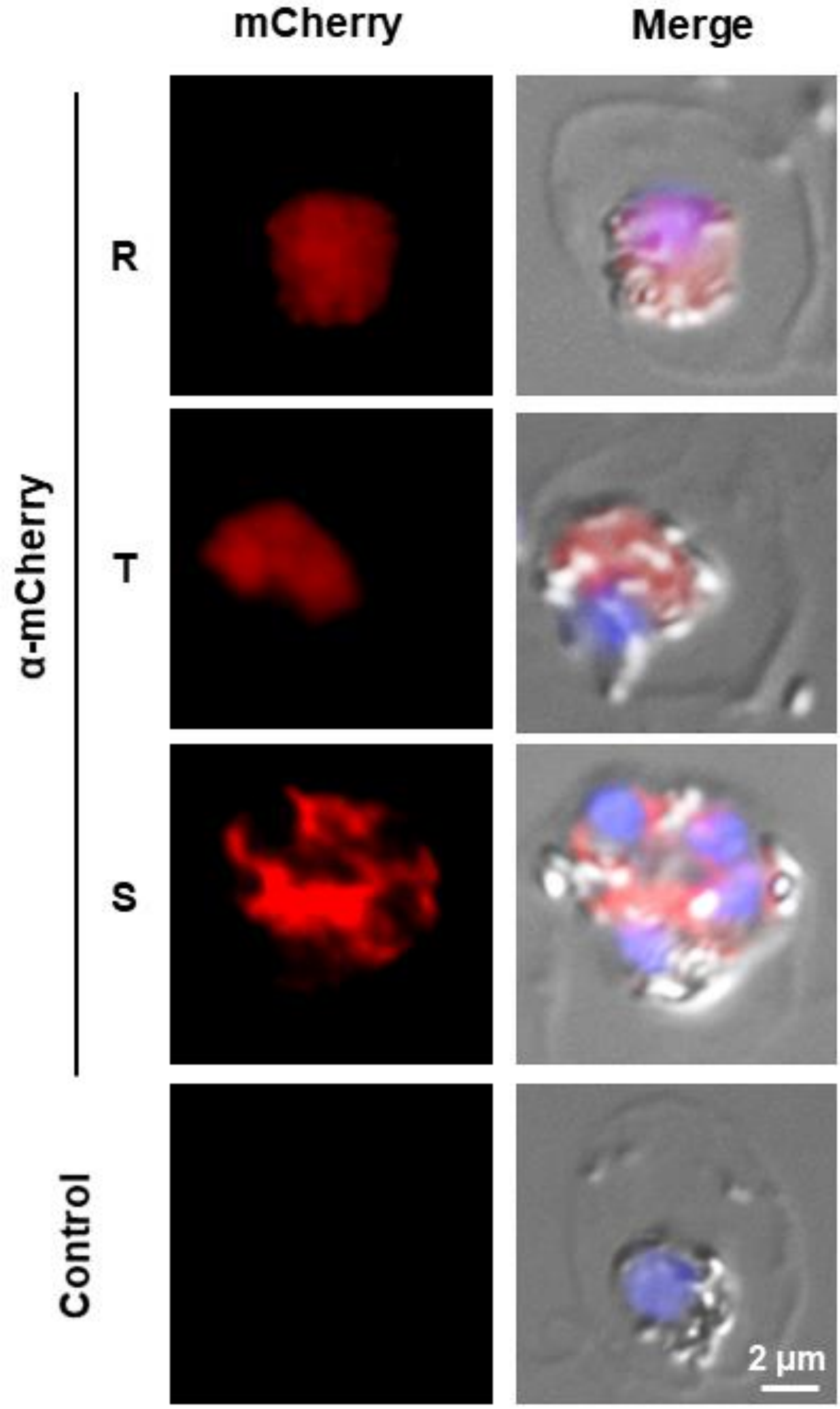

Fig. S7. Representative IFA images of mCherry transgenic *P. knowlesi*-infected monkey RBCs. Parasites were stained with anti-mCherry antibody (α-mCherry, red). the α-mCherry-stained image was merged with DAPI nucleus-staining (blue) and differential interference contrast (DIC) images (Merge). The bottom panels are from a negative control reacted with normal rabbit IgG.

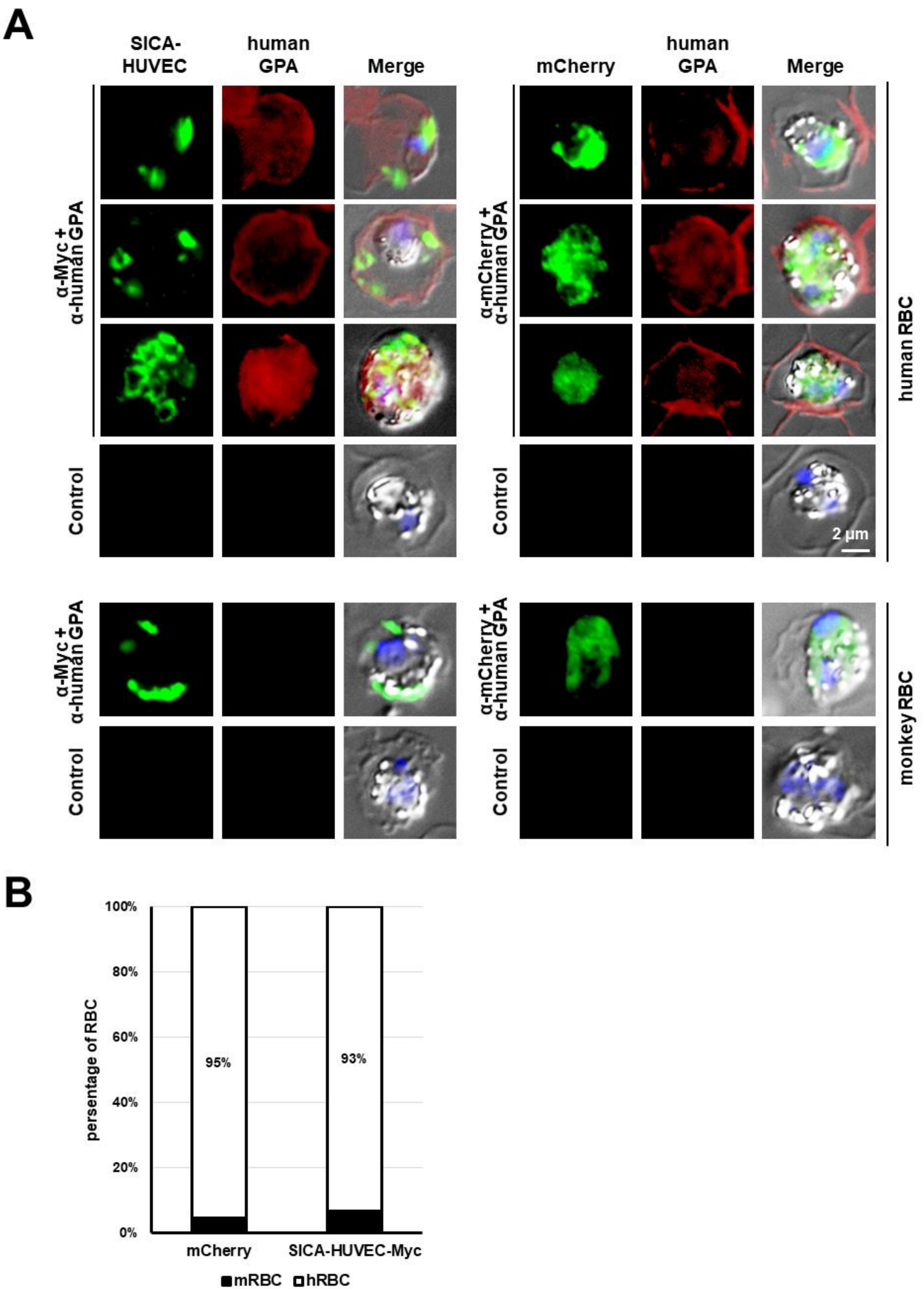

Fig. S8. (A) Representative IFA images of human (upper panel group) or monkey (lower panel group) RBCs infected with a transgenic *P. knowlesi* line expressing SICA-HUVEC-Myc (left panel group) or mCherry (right panel group). Smears were stained with mouse anti-Myc antibody (α-Myc, green) or rabbit anti-mCherry antibody (α-mCherry, green), rat anti-human glycophorin A antibody (α-GPA, red), and DAPI (nucleus, blue). Images were merged with differential interference contrast (DIC) image (Merge). The bottom panels of each group are from a negative control reacted with normal mouse IgG or normal rabbit IgG and normal rat IgG. (B) Ratio of monkey and human RBCs infected with transgenic *P. knowlesi* line expression mCherry (left) or SICA-HUVEC-Myc (right).

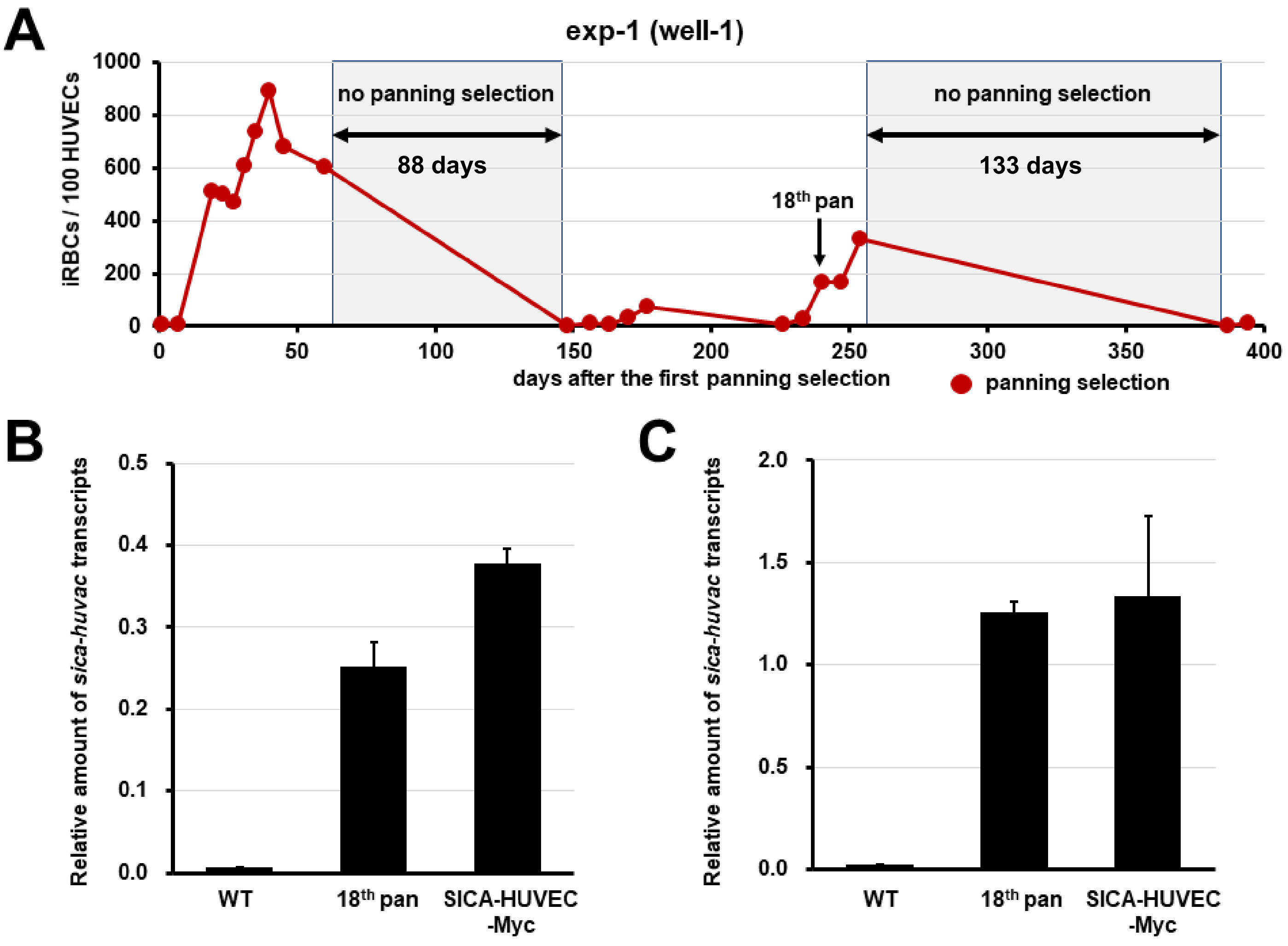

Fig. S9. Relative amount of transcripts of *sica-huvac* normalized by *methionine tRNA ligase* transcripts by qRT-PCR. (A) The time course of the first panning selection experiment of *P. knowlesi*-iRBCs against HUVECs. Panning selection was restarted 88 days after the 10th pan shown in figure 1A and the RNA sample of the 18th pan was obtained, when iRBC binding activity was 168.4 iRBCs/100 HUVECs. In this experiment, the binding activity of iRBCs was assessed 88 days (left) or 133 days after the panning selection and no binding activity was seen. *sica-huvec* transcripts were amplified with primers SICA-HUVEC.rt-F1 and SICA-HUVEC.rt-R1 (B) or SICA-HUVEC.rt-F2 and SICA-HUVEC.rt-R (C). Primer information is in Table S1. RNA was obtained from wild-type *P. knowlesi* DMU line (WT), the 18th pan of WT line described above (18th pan), and *P. knowlesi* SICA-HUVEC-Myc-expressing *P. knowlesi* (SICA-HUVEC-Myc) in monkey RBCs.

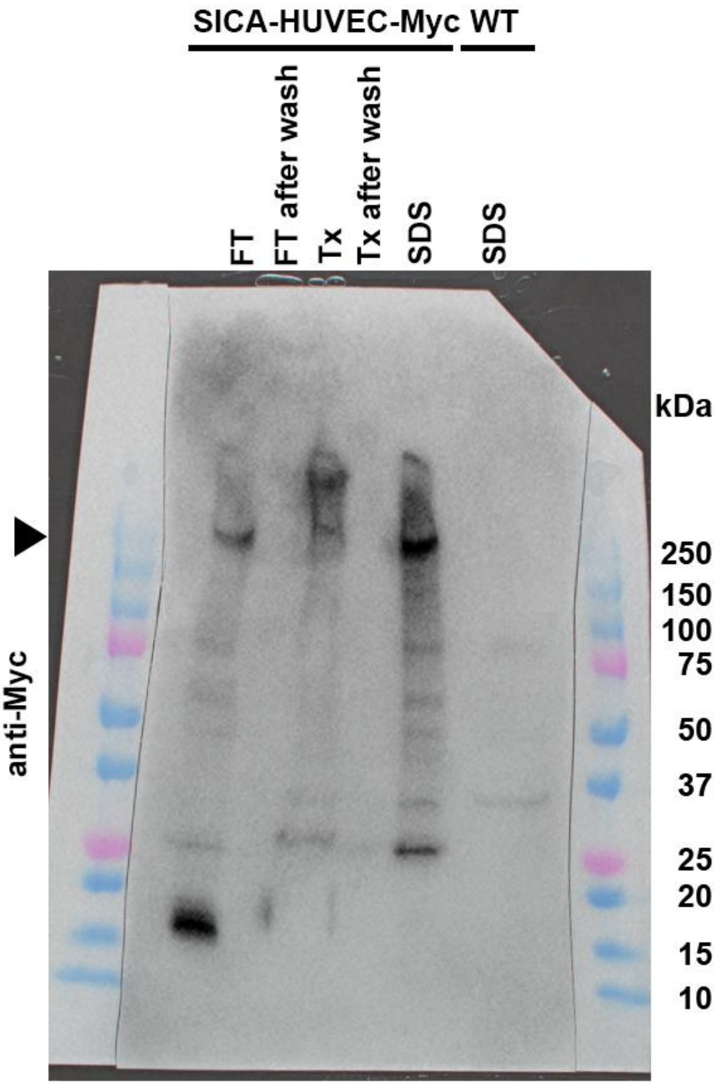

Fig. S10. The original blot of control of Fig. 3B without cropping. Western blotting of the wild-type parental *P. knowlesi* line (WT) and the transgenic line expressing SICA-HUVEC-Myc in monkey RBCs. Proteins were sequentially extracted by freeze-thawing (FT), with 1% Triton X-100 (Tx), and then with 2% SDS for the transfectants; whereas they were extracted with 2% SDS only for the wild-type parasites. Bands detected with anti-Myc antibody (anti-Myc) around the expected size for the SICA-HUVEC-Myc are indicated with an arrowhead.

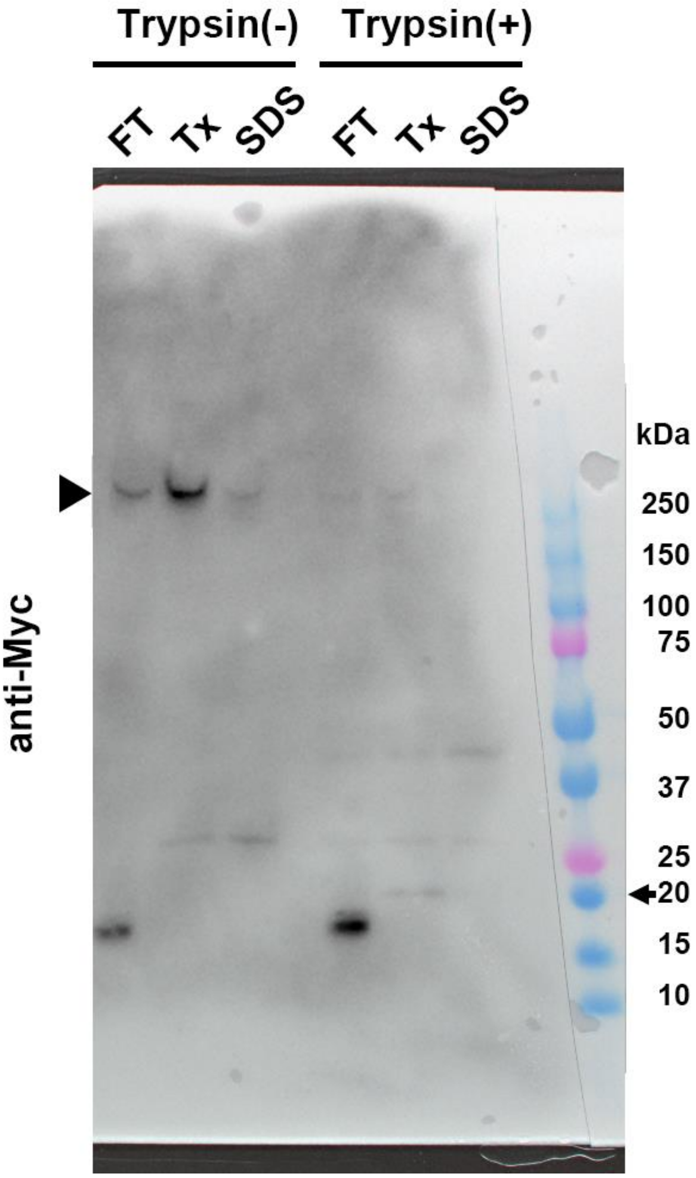

Fig. S11. The original blot of Fig. 3C top (stained with anti-Myc antibody) without cropping. Trypsin treatment of the transgenic line expressing SICA-HUVEC-Myc. Trypsin-treated or untreated samples were sequentially extracted and subjected to Western blot with anti-Myc antibody (anti-Myc). Bands detected with anti-Myc antibody around the expected size for the full-length of SICA-HUVEC-Myc are indicated with an arrowhead. A band at 24 kDa is indicated with an arrow.

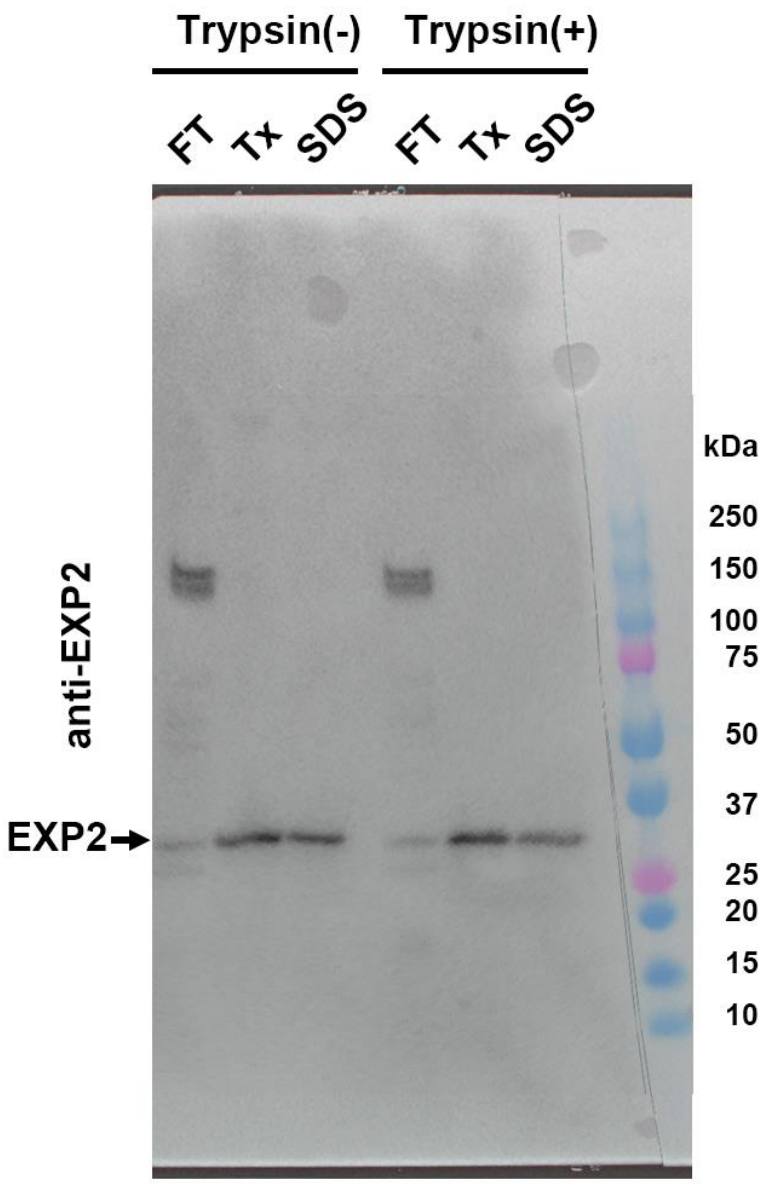

Fig. S12. The original blot of Fig. 3C bottom (stained with anti-EXP2 antibody) without cropping. Trypsin treatment of the transgenic line expressing SICA-HUVEC-Myc. Trypsin-treated or untreated samples were sequentially extracted and subjected to Western blot with anti-EXP2 antibody (α-EXP2). The expected EXP2 band (EXP2) is indicated with an arrow, which is consistent with the calculated molecular weight 31.9 kDa of *Plasmodium knowlesi* EXP2 (PKNH_1209900). A band in the FT fraction at approximately 150 kDa is a possible homomultimer of EXP2.
